# Repurposing drugs with specific activity against L-form bacteria

**DOI:** 10.3389/fmicb.2023.1097413

**Published:** 2023-04-04

**Authors:** Kaveh Emami, Peter Banks, Ling Juan Wu, Jeffery Errington

**Affiliations:** ^1^Centre for Bacterial Cell Biology, Faculty of Medical Sciences, Newcastle University, Newcastle upon Tyne, United Kingdom; ^2^Faculty of Medical Sciences, Biosciences Institute, Newcastle University, Newcastle upon Tyne, United Kingdom

**Keywords:** bacterial cell wall, L-form bacteria, FDA drug-library, calcium channel blockers, dihydropyridines, diphenylmethylpiperazine, manidipine, membrane fluidity

## Abstract

Cell wall deficient “L- form” bacteria are of growing medical interest as a possible source of recurrent or persistent infection, largely because of their complete resistance to cell wall active antibiotics such as β-lactams. Antibiotics that specifically kill L-forms would be of potential interest as therapeutics, but also as reagents with which to explore the role of L-forms in models of recurrent infection. To look for specific anti-L-form antibiotics, we screened a library of several hundred FDA-approved drugs and identified compounds highly selective for L-form killing. Among the compounds identified were representatives of two different classes of calcium channel blockers: dihydropyridines, e.g., manidipine; and diphenylmethylpiperazine, e.g., flunarizine. Mode of action studies suggested that both classes of compound work by decreasing membrane fluidity. This leads to a previously recognized phenotype of L-forms in which the cells can continue to enlarge but fail to divide. We identified a considerable degree of variation in the activity of different representatives of the two classes of compounds, suggesting that it may be possible to modify them for use as drugs for L-form-dependent infections.

## Introduction

The cell wall is a major structural feature of virtually all bacteria that is normally essential for viability (Egan et al., 2020). Consequently, it has long been the target for favored antibiotic drugs, such as the β-lactams, including penicillins, cephalosporins, monobactams, and others. In typical bacterial culture media, which are generally of relatively low osmolarity, wall deficient cells can undergo catastrophic lysis due to loss of the osmoprotective action of the tough elastic wall. However, in iso-osmotic conditions, such as those in many human tissues and fluids (e.g., urine), loss of the cell wall can be tolerated, and in some cases the cells can grow in a wall-deficient state called the L-form (Allan et al., 2009; Errington et al., 2016). L-forms are remarkable in several ways, not least that their proliferation occurs by budding or vesiculation mechanisms that are independent of the normally essential FtsZ-dependent division machine (Leaver et al., 2009; Mercier et al., 2014; Studer et al., 2016).

Recent years have seen a renewal of interest in L-form bacteria from both a basic science perspective and as a possible source of recurrent infection (Dell’Era et al., 2009; Leaver et al., 2009; Mercier et al., 2013; Studer et al., 2016; Kawai et al., 2018; Ramijan et al., 2018; Mickiewicz et al., 2019; Wu et al., 2020). Although L-forms are intrinsically more fragile than walled cells, and require isotonic conditions to avoid lysis, they can survive and are likely to grow to an extent in various inter- and intracellular niches of infected patients (Domingue, 2010; Errington et al., 2016; Mickiewicz et al., 2019). They can therefore survive and potentially grow during prolonged periods of treatment with cell wall active antibiotics, to which they are completely resistant. While there are numerous reports of L-forms in association with a wide range of infectious diseases (Domingue and Woody, 1997; Domingue, 2010), direct evidence for their role in pathogenesis or persistence is largely lacking. A key problem is that the bacteria responsible for recurrence following antibiotic treatment are inevitably rare, so it is difficult to demonstrate that L-forms and not the various other forms of persister cells, are responsible (Clasener, 1972; Onwuamaegbu et al., 2005; Allan et al., 2009; Errington et al., 2016; Lazenby et al., 2022).

As a possible route toward testing the L-form hypothesis of recurrence and, potentially to new antibiotics capable of eliminating L-form persistence, we ran a screen for low MW drug-like compounds that specifically kill L-form bacteria. This strategy has previously been used to identify drugs acting on intracellular pathogens (Czyz et al., 2014). Several hundred compounds from a library of U.S. Food and Drug Administration (FDA) approved drugs were screened for differential activity on walled and L-form *Bacillus subtilis*. We subsequently focused on two families of calcium channel blockers in which some but not all analogs tested have specific activity against L-forms. The potency and specificity of some of the compounds, and the availability of preliminary information on structure-activity relationships, suggests that it will be possible to develop effective L-form-targeting drugs.

## Results

### A screen for specific inhibitors of *B. subtilis* L-forms

We developed a screen for specific inhibitors of L-forms over isogenic walled cells that could be run in high-throughput mode. We used a stable L-form strain of *B. subtilis* based on a spontaneous 18 kbp deletion described previously by Mercier et al. (2013). Although the original strain was lost, we had a frozen chromosomal DNA sample and were able to recover the mutation into our wild type *B. subtilis* lab strain 168CA. This was done by transformation and selection for growth in the presence of 250 μg/ml penicillin G. The resultant strain was subject to whole genome sequencing, which confirmed the presence of the correct 18 kbp deletion (Supplementary Figure 1). The deletion removes the *murC* gene required for peptidoglycan precursor synthesis (van Heijenoort, 2007; White et al., 2010) and one or more genes, as yet undefined, that enable the cells to grow in the L-form state by protecting them from oxidative damage (Kawai et al., 2015; Kawai et al., 2019).

**FIGURE 1:**
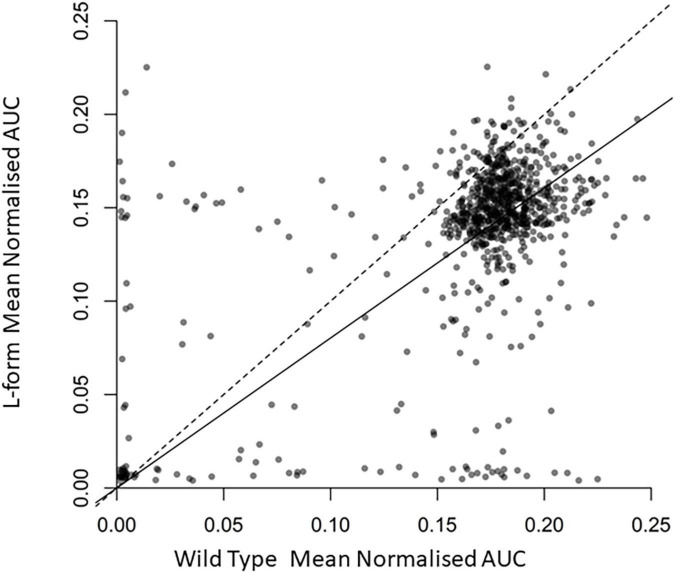
Effects of the U.S. Food and Drug Administration (FDA) drug library compounds on the growth of L-form and walled cells. Scatter plot displaying normalized area under growth curve for wild type *B. subtilis* against L Form cultures grown in the presence of around 800 different compounds. Each point represents the relevant strain grown in culture containing a different compound. Transparency of each point is set to 50% to display the density of the plot. The dashed line is the one to one line representing equal growth, the solid line represents the line of best fit from a linear regression model forced through the zero axis point. Full data are provided in Supplementary Table 1.

We tested various growth conditions to enable efficient and reproducible growth of both the L-form and its isogenic walled parent strain (see methods). We then used these conditions to screen a collection of several hundred FDA-approved drugs (Selleckchem).^1^ Each compound was tested at a concentration of 10 μM in quadruplicate. The results of two independent screening runs are given in Supplementary Tables 1, 2. Overall, the results of the two screens were similar. The average values for growth of walled and L-form strains were calculated and the results of the first experiment are plotted in Figure 1.

As expected, most compounds had no effect on growth of either strain, and their points therefore appear as a cloud toward the top right of the plot. Compounds to the bottom left inhibited growth of both strains at the concentration used. Compounds to the top left and bottom right were differentially active, selectively inhibiting the growth of walled cells or L-forms, respectively. Walled-cell-specific compounds mainly included antibiotics known to be active on cell wall targets, e.g., several beta-lactams and vancomycin, consistent with expectation for the screening approach used.

### Two families of calcium channel blocker drugs specifically inhibit L-form growth

**FIGURE 2:**
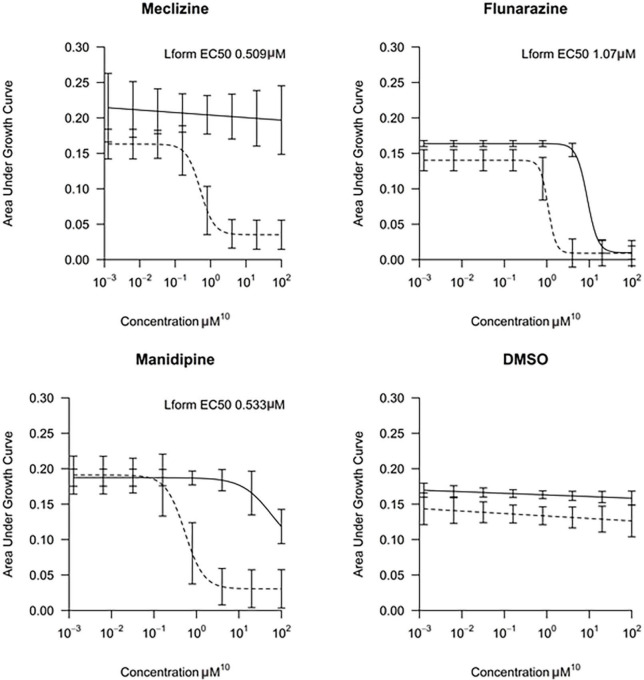
Dose-response tests of potential L-form specific compounds. Growth of wild type (solid line) and L-form (dashed line) as measured by area under the growth curve was plotted against concentration in response to three different compounds and DMSO. The error bars represent the 95% confidence level.

The top L-form-specific hit in the second screen, manidipine, was of particular interest because it belongs to a large family of drugs with closely related chemical structures, sharing a dihydropyridine (DHP) nucleus, several of which were present in the screening set. Table 1, columns 2 and 3, show that these compounds varied substantially in differential activity, suggesting that the series might provide structure-activity information on the L-form killing effect. To investigate this in more detail, we obtained resupplies of the commercially available compounds from the original tests, as well as several compounds not in the FDA collection, and tested them in dose response mode to obtain MIC values for walled and L-form cultures (Table 1, columns 3 and 4). Table 1 column 5 shows the magnitude of the difference between killing on L-forms vs. walled cells, and the compounds are ranked according to this difference value. Figure 2 shows typical examples of the dose response assays.

**TABLE 1:** Differential effects of selected compounds.

Compound	Library screening		Dose response			Structure	MW*	ACD/LogP*
	**Residuals, exp. 1**	**Residuals, exp. 2**	**Walled EC_50_**	**L-form EC_50_**	**Difference walled/L-form**			
**Dihydropyridines**								
Manidipine	–0.14	–0.14	>100	0.53	>190	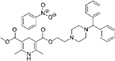	610.7	5.46
Lacidipine	0.021	0.016	>100	0.58	>170	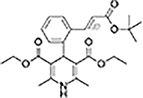	455.5	5.49
Lercanidipine	ND	ND	>100	0.58	>170	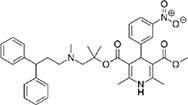	611.7	8.04
Cilnidipine	–0.064	–0.076	>100	1.8	>56	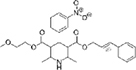	492.5	5.36
Azelnidipine	0.023	0.016	>100	0.2	>50	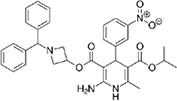	582.6	4.21
Benidipine	ND	ND	>100	2	>50	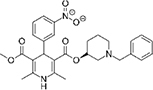	505.6	4.92
Nimodipine	0.014	–0.0046	>100	2.3	>43	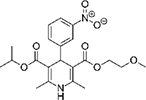	418.4	3.86
Nicardipine	ND	ND	>100	6.8	>15	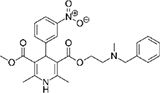	479.5	5.13
Clevidipine	–0.0039	–0.011	22	4.5	4.9	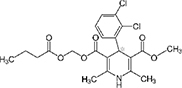	456.3	5.46
Nitrendipine	0.0022	–0.024	>100	21	>4.8	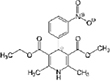	360.4	3.50
Isradipine	–0.015	–0.052	>100	40	>2.5	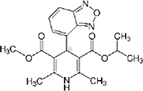	371.4	3.59
Amlodipine	0.0036	0.00013	>100	46	>2.2	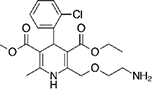	408.9	4.16
Felodipine	–0.046	–0.036	6.1	4.5	1.4	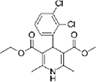	384.3	4.83
Nifedipine	ND	ND	>100	100	1	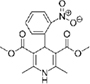	346.3	2.97
Nisoldipine	0.0059	0.029	>100	>100	1	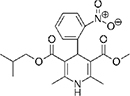	388.4	4.38
Nilvadipine	0.027	0.027	38	48	0.79	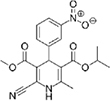	385.4	1.72
**Diphenylmethylpiperazines**								
Meclizine	–0.13	–0.13	>100	0.51	>200	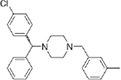	390.9	4.99
Flunarizine	–0.098	–0.13	8.9	1.1	8.1	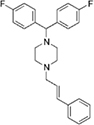	404.5	4.74
Cetirizine	0.013	–0.011	>100	>100	1	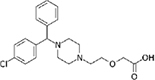	388.9	2.16

*From the ChemSpider web site (chemspider.com).

The compounds varied substantially in both potency and specificity. In general, they had no significant effect on walled cells, so the variation was mainly in relation to their effects on L-form growth. Manidipine was consistently one of the most potent and selective compounds. Surprisingly, azelnidipine and lacidipine, which were essentially inactive in the library screening, were both as potent and selective as manidipine. It seems possible that the library compounds had degraded during storage. A fourth compound, lercanidipine, not present in the library screen also showed high potency and selectivity. Amlodipine and benidipine were less potent but still selective. The remaining DHP compounds tested did not show a significant degree of potency for either L-forms or walled cells. Comparisons of compound structure and activity suggested that higher MW and greater hydrophobicity are important for the L-form-specific activity, and three of the four most potent compounds also possessed a nitrobenzene moiety, although this was also present in some of the inactive compounds.

The DHPs are calcium-channel blockers (CCBs) used as anti-hypertensive drugs. We noticed that meclizine and flunarizine, representative of a different chemical class of CCBs, the diphenylmethylpiperazines (DPMPs), also scored highly in the primary screen, so we procured and tested three of these compounds. The results (Figure 2 and Table 1) showed that two closely related compounds, flunarizine and meclizine both had very strong and selective activity against L-forms, similar to that of manidipine. In contrast, the related compound cetirizine was inactive. Cetirizine differs prominently from the others by the presence of an ether linked acetate group, so it seems likely that this charged group is responsible for the lack of activity of cetirizine.

### Manidipine and flunarizine inhibit the division of L-form cells

**FIGURE 3:**
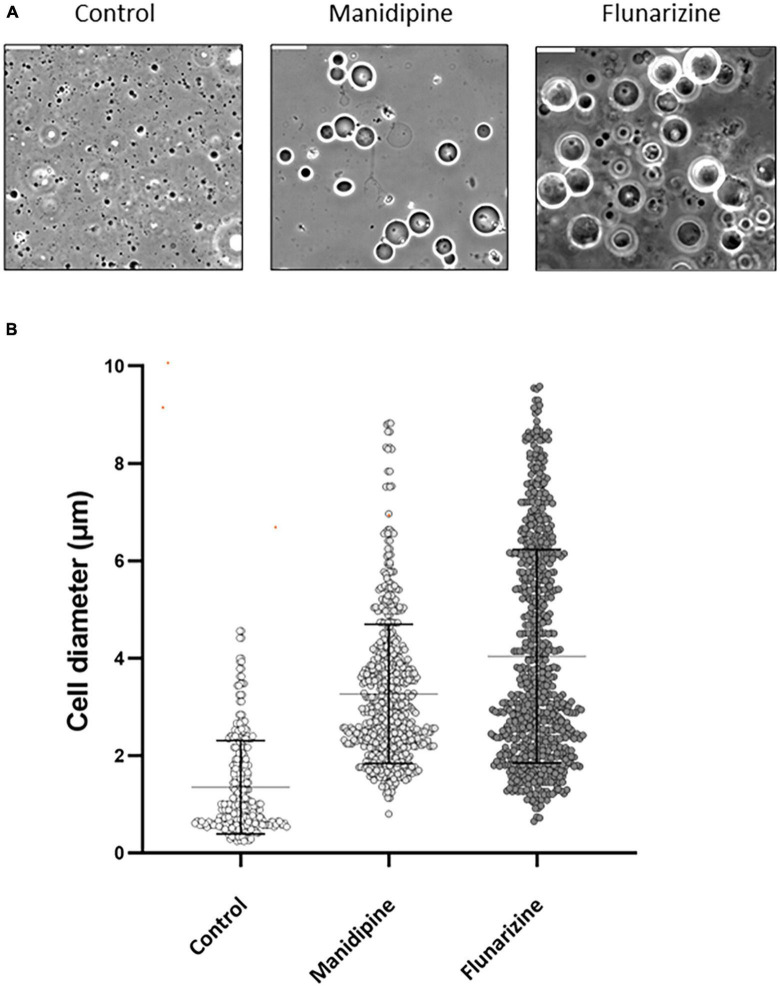
Morphological effects of manidipine and flunarizine on L-form cells. The L-form strain was grown in MSM and parallel cultures were grown for 12 h, untreated (control) or in the presence of 25 μM manidipine or 12 μM flunarizine. **(A)** Typical fields of cells visualized by phase contrast microscopy. Scale bar = 10 μm. **(B)** The diameters of >600 cells from each treatment were measured using Fiji image processing package. Mean and standard error values are indicated by solid black lines.

We chose manidipine and flunarizine, as representatives of the DHP and DPMP compound series, for further investigation. To better understand their mode of action we used phase contrast imaging to follow the fate of L-forms when cultured in a liquid medium in the presence of an inhibitory concentration of each of the compounds. Under the conditions used the untreated L-form cells had their characteristically heterogeneous appearance, with great variation in size and shape, and also the presence of abundant blebs and tubules that are associated with cell division events (Leaver et al., 2009; Figure 3A, control). After culture for 15 h in the presence of manidipine or flunarizine, relatively few cells were evident compared to the untreated cells, but those that were present were large and almost spherical (Figures 3A, B). Examination of time lapse imaging experiments suggested that generation of the large spherical cells was a result of impaired division Supplementary Video 1). Thus, the cells were able to enlarge to a significant extent, but division was inhibited by the drugs.

To test whether the cell enlargement was a terminal phenotype, as for L-forms with the genetic defect in membrane fluidity (Mercier et al., 2012), we treated L-form cultures with inhibitory concentrations of manidipine or flunarizine and, after allowing them to accumulate in the enlarged state, diluted the cultures to essentially remove the compounds. In Supplementary Figure 2 dotted lines show the undiluted cultures. Green and blue lines show the growth inhibitory effects of two different concentrations of manidipine (green) and flunarizine (blue), relative to the DMSO only control (black). After 22 h, a sample of each culture was diluted 20-fold and growth at greatly reduced inhibitor concentration was followed. Cells cultured at the higher concentration of flunarizine (20 μM) showed no recovery over nearly 20 h. The other diluted cultures all showed a significant delay before resuming growth. These results suggest that a sub-population of cells (probably the grossly enlarged cells) are unable to resume growth following inhibitor treatment.

### Laurdan fluorescence assay for membrane fluidity

**FIGURE 4:**
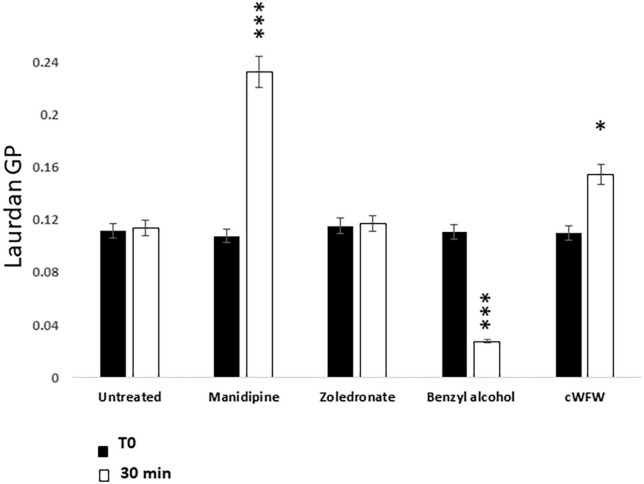
Effect of Manidipine on membrane fluidity as measured by Laurdan fluorescence. Laurdan GP values were measured for untreated cells, and for cells incubated with test compounds manidipine (25 μM), or membrane fluidizer benzyl alcohol (50 mM). The cyclic hexapeptide cWFW was used (12 μM) as a negative control (reduces membrane fluidity). *P*-values ^***^p < 0.001, *p < 0.05. Means ± SD of five technical replicates.

We previously described a genetic screen for *B. subtilis* mutants able to grow in the walled state but not as L-forms (Mercier et al., 2012). The best characterized mutation affected synthesis of the precursors to branched-chain fatty acids, leading to a decrease in membrane fluidity. The mutant cell phenotype was very similar to that produced by treatment with manidipine. The cells enlarged, occasionally underwent shape changes characteristic of dividing L-forms but then the final scission step needed to generate separated daughter cells was blocked. Given these similarities we wondered if manidipine and flunarizine worked by reducing membrane fluidity. To test this we used a fluorescence assay based on Laurdan Dye (6-Dodecanoyl-2–dimethylaminonaphthalene) (Parasassi et al., 1990). Unfortunately, flunarizine is intrinsically fluorescent in the wavelengths needed for the assay, so we were able to run this assay only for manidipine. L-form cells were cultured with or without manidipine (25 μg ml^–1^) and then treated with the Laurdan dye. For controls, we treated parallel samples of L-forms with benzyl alcohol or the membrane active peptide cWFW, which are known to increase or decrease membrane fluidity, respectively (Scheinpflug et al., 2017b). We also tested zoledronic acid, a compound that showed differential activity in the primary screening (Supplementary Table 2) but is chemically quite different from the CCBs in being highly charged. As shown in Figure 4, zoledronic acid had no significant effect on fluidity, compared with the untreated culture, while the control compounds decreased (benzyl alcohol) or increased (cWFW) Laurdan fluorescence, as expected. Importantly, manidipine addition resulted in a substantial increase in Laurdan fluorescence, indicative of a decrease in membrane fluidity (Scheinpflug et al., 2017a). These results suggest that manidipine interferes with L-form division by decreasing membrane fluidity, thereby blocking the scission step in the division.

### Genetic assay for effects on membrane fluidity

*Bacillus subtilis* controls membrane fluidity by altering two structural features of its fatty acids: methylation at the distal end of the carbon chain, to create branched chain fatty acids; and insertion of double bonds, *via* the action of a desaturase enzyme, encoded by the *des* gene. Both modifications tend to increase membrane fluidity by impairing the packing of alkyl chains. Strain HS527 has deletions Δ*bkd* and Δ*des* that eliminate both sources of membrane fluidization (Gohrbandt et al., 2022). Branched chain fatty acid synthesis can be restored in cells bearing the Δ*bkd* mutation by supplying branched chain fatty acid precursors (Willecke and Pardee, 1971; Mercier et al., 2012). Supplementation with isobutyrate (IB) or isovalerate generate iso-BCFAs, which have relatively little effect on membrane fluidity, whereas 2-methylbutyrate (MB) generates ante-iso-BCFAs, which increases fluidity. Reduced membrane fluidity results in impaired growth of walled cells (and L-forms) at lower culture temperatures. If manidipine and flunarizine worked by reducing membrane fluidity, cells of the Δ*bkd Δdes* mutant should show impaired growth at lower temperatures and this impaired growth should be rescued by supplementation with MB but not IB.

As shown in Table 2, cells cultured at 30^°^C in the presence of non-fluidizing IB showed a significantly reduced growth rate compared to untreated cells or cells treated with zoledronate, as a chemically distinct control. That this was due to an effect on membrane fluidity was confirmed by the restoration of growth rate in the presence of the fluidizing precursor MB.

**TABLE 2:** Effects of compounds on growth (doubling time; h) of the Δ*bkd Δdes* mutant at 30^°^C in the presence of fluidizing (MB) and non-fluidizing (IB) precursors.

	MB	IB	*P*-value
Untreated	1.5 ± 0.04	1.5 ± 0.08	N.S.
Manidipine	1.4 ± 0.06	1.8 ± 0.08	<0.001
Flunarizine	1.4 ± 0.07	2.39 ± 0.14	<0.0001
Zoledronic acid	1.5 ± 0.06	1.6 ± 0.15	N.S.

Mean ± SD of five technical replicates. *P*-values based on an unpaired two-sided *t*-test. N.S., non-significant.

### DHPs are active on L-forms of the pathogen, *Enterococcus faecalis*

**FIGURE 5:**
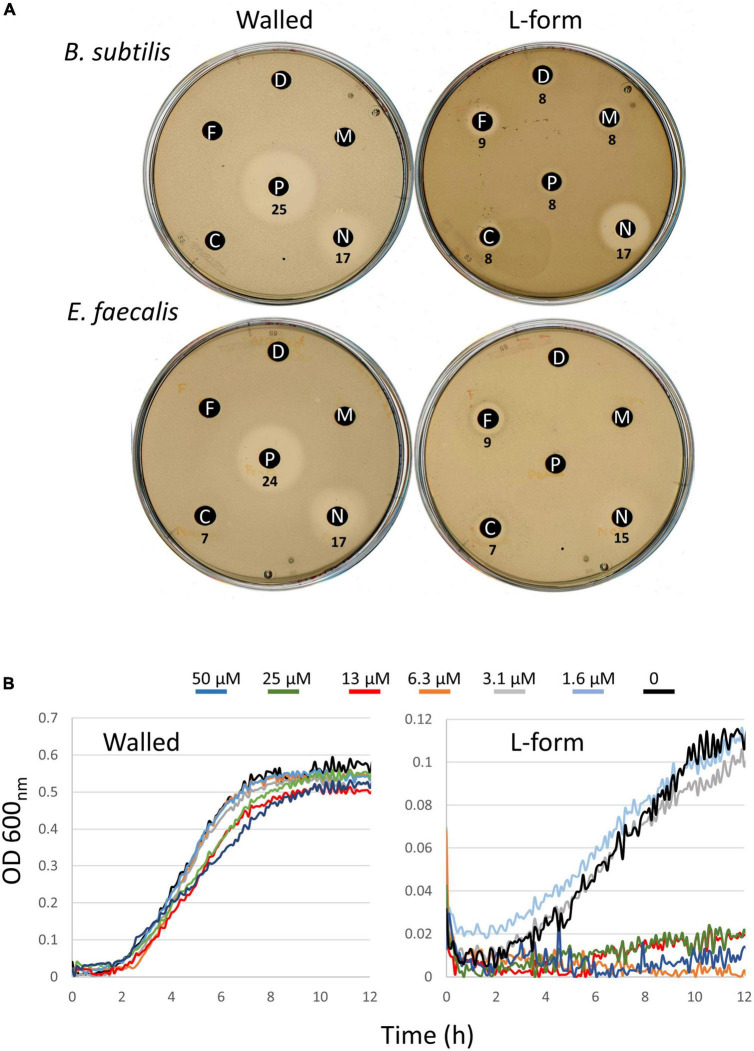
Differential effects of compounds on walled cells and L-forms of *E. faecalis*. **(A)** Disk diffusion tests on walled and L-form strains of *B. subtilis* (upper) and *E. faecalis* (lower). Antibiotic abbreviations and amounts added to discs as follows. C, meclizine (50 μg); D, DMSO (12 μl); F, flunarizine (50 μg); M, manidipine (100 μg); N, novobiocin (60 μg); P, penicillin G (40 μg). Diameters of the zones of inhibition (including the 6 mm paper disks) are shown in mm. **(B)** Differential effects of meclizine on growth of walled (left) and L-form (right) cultures of *E. faecalis*. Diameters of the zones of inhibition (including the 6 mm paper disks) are shown in mm.

Finally, it was interesting to test whether the compounds specifically killing *B. subtilis* L-forms also worked on L-forms of pathogenic organisms. We recently found that a strain of *E. faecalis* was relatively easy to shift into the L-form state and proliferate efficiently. It was therefore interesting to test whether this strain was also susceptible to the compounds described above. Unfortunately, *E. faecalis* grew quite poorly under the conditions used to screen against *B. subtilis* in liquid medium. We therefore turned to an alternative disc diffusion method for examining susceptibility. This method was also somewhat limited as, although control compounds PenG and Novobiocin gave large haloes, with PenG, as expected, being highly selective for the walled cells, those of the compounds active on *B. subtilis* were relatively small (Figure 5A). Nevertheless, it was evident that the two DHP compounds flunarizine and meclizine, were both specifically active on the *E. faecalis* L-forms. Manidipine did not give a clear inhibition on either the walled or L-form cultures, although activity on *B. subtilis* L-forms was also difficult to discern under these conditions. We suspect that the haloes are small because the compounds are quite hydrophobic and do not diffuse readily through agar. In the light of these results, we returned to the liquid assays. Despite the slow and poorly reproducible growth of the *E. faecalis* L-form strain in liquid culture, in a series of repeats we were able to show that the L-form was sensitive at least to meclizine, with at least an ∼10-fold difference in growth inhibitory concentrations (6.3 μM vs. >50 μM; Figure 5B).

## Discussion

L-forms have long been discussed as a likely source of recurrence in infectious disease, due to their ability to tolerate and indeed grow in the presence of normally lethal concentrations of cell wall active drugs (Domingue, 2010; Westblade et al., 2020). Nevertheless, their clinical significance remains unclear and somewhat controversial, mainly because direct evidence for L-forms as the source of recurrence following antibiotic treatment is lacking. Availability of an L-form specific inhibitor would enable experiments to test whether specific elimination of L-forms would prevent recurrence. Furthermore, such compounds would then provide potential clinical agents with which to prevent or reduce recurrence in patients. We set out to find such compounds by screening a library of existing drugs and were able to identify a substantial number of compounds that are much more inhibitory on L-forms than walled cells of *B. subtilis*. Further work is needed to systematically explore all the potential hits, but here we identify and describe two particularly interesting chemical classes of drug, each of which encompasses compounds with good potency and specificity. In both cases, availability of a range of clinically validated compounds allowed us to obtain some preliminary structure-activity relationship (SAR) data. These data suggest that both the DHP and DPMP series of drugs may be amenable to further medicinal chemistry with the aim of retaining strong selective activity against L-forms, while reducing or eliminating the potentially deleterious “side effects” associated with the existing therapeutic activities of the drugs. Importantly, both classes of drug have been extensively used clinically (see Wishart et al., 2018 and associated Drugbank database), (Wishart et al., 2018) and have excellent safety profiles, so development of analogs as L-form specific agents could be of relatively low risk in terms of pharmacology and toxicity.

Meclizine appears particularly interesting as the starting point for a drug. It has been used clinically as both an antihistamine and an anti-motion-sickness drug (Patel and Ambizas, 2011). It is well-tolerated, with typical dosing up to 50 mg per day (Drugbank), only about one order of magnitude less than the typical dosing for antibiotics (McKenzie, 2011). The low IC_50_ value (0.51 μg/ml) against L-forms is already typical of antibiotics in the clinic, suggesting again the potential for developing a drug from this compound series. Importantly, it appears that meclizine and flunarizine, at least, are also active against L-forms of the pathogen *E. faecalis*, as well as *B. subtilis*.

*B. subtilis* L-forms treated with manidipine or flunarizine continued to grow for a while but division appeared to be impaired, leading to an increase in average cell size but a reduction in cell number, compared to the untreated controls. We previously showed that division of *B. subtilis* L-forms largely occurs by a remarkable “vesicle scission” process that is completely independent of the complex FtsZ-dependent machine used for division by most walled bacteria (Leaver et al., 2009; Mercier et al., 2014). The block in division followed by increased cell size was reminiscent of the phenotype of mutants that we previously isolated that are specifically blocked for L-form growth (Mercier et al., 2012). Characterization of these mutants, which carried mutations in the *lpdV* or *bkd* genes suggested that the block in L-form division was due to reduced membrane fluidity (Mercier et al., 2012). Three lines of evidence, based on cell morphology, biochemistry, and genetic sensitization, suggested that manidipine (a DHP), and probably flunarizine (a DPMP) also work by reducing membrane fluidity, consistent with the notion that L-forms have a specific requirement for higher membrane fluidity, likely impacting on the scission step in L-form division. Although we have not ruled out the possibility that the compounds work by inducing regulatory changes in membrane composition, we think this unlikely due to the rapidity with which the inhibition of cell division takes place.

The DHP and at least some DPMP drugs have been reported to affect calcium dynamics or homeostasis, through interactions with calcium channels or G-protein coupled receptors (Drugbank), but it is by no means clear what are the specific targets of each of the drugs. This is reflected in the different clinical indications for which they are used. For example, manidipine is used in the treatment of hypertension, flunarizine predominantly for migraine, meclizine for motion sickness and cetirizine as an antihistamine (Drugbank). It is therefore not easy to deduce whether their action against L-forms might involve calcium channel activity. Also, rather little is known about calcium homeostasis in bacteria. Where putative Ca channels have been studied in well-characterized organisms, it does not seem as though they perform an essential role, although this would not rule out a specific effect on L-forms.

From the perspective of possible development of these compounds as drugs it will be important to understand whether their action is bacteriocidal or bacteriostatic. We know that mutations that reduce membrane fluidity in L-forms are lethal and result in accumulation of enlarged cells that fail to divide. Our preliminary experiments (e.g., Supplementary Figure 2) support the idea that the compounds are killing a subset of cells but more work will be needed to test our hypothesis that the cell enlargement is indeed a terminal state.

For the future, we are aware that a substantial number of other compounds were specifically affected in L-form growth. Many of the compounds are quite hydrophobic (see logP values in Table 1), suggesting that they may also work *via* effects on membrane fluidity but this remains to be investigated. However, our main focus will be to evaluate whether the DHP or DMPM compounds identified as specific inhibitors of L-forms can be used to test the role of L-forms in models of recurrent infection, such as that recently described by Ganguli et al. (2022). While this work was in progress we serendipitously found that a novel polyene compound called demurilactone also differentially kills L-forms of *B. subtilis* (Dashti et al., 2022).

## Materials and methods

All experiments were performed more than two times.

### Chemicals and media

All chemicals, unless stated otherwise, were from Merck.

Liquid medium used for *B. subtilis* growth was nutrient broth (NB, Oxoid). Osmoprotective medium for screening was MSM, which is: 0.5 M sucrose, 40 mM maleic acid, and 40 mM magnesium chloride. For the assays with liquid medium in multi-well plates, a 1:1 mixture of NB/MSM was used.

### Bacterial strains

The 168CA strain was the laboratory stock strain. The L-form strain (Δ18) was generated using genomic DNA from Mercier and co-workers (Mercier et al., 2012). *Bacillus subtilis* 168CA cells were made competent for transformation with DNA either by the method of Hamoen et al. (2002) or that of Anagnostopoulos and Spizizen (Anagnostopoulos and Spizizen, 1961) as modified by Jenkinson (1983).

Strain HS527 (Δ*bkd::erm*, Δ*des::spc*) was donated by Dr. H. Strahl.

### Genome sequencing and analysis

Genomic DNA of the original Δ18 strain was prepared using Monarch^®^ Genomic DNA Purification Kit (New England Biolabs). Genomic DNA sequencing was done by the NU-OMICS DNA sequencing research facility, Northumbria University, Newcastle upon Tyne, using Illumina NexteraXT technology. Genome *de novo* assembly and analysis were done using the QIAGEN CLC Main Workbench software version 11.0.2 (CLC Bio, Qiagen, Valencia, CA, United States).

### U.S. Food and Drug Administration library screening

The FDA compound library was obtained from Selleckchem (see text foot note 1). The library contained 760 approved drugs, all at 10 mM in 100% DMSO. Cultures of L-form or 168CA grown in MSM-NB medium were diluted 1 in 100 or 1 in 10 into fresh medium and distributed into 96 well flat bottom plates (Greiner Bioone 655161). Single compounds were added to each well using a Beckman Biomek FX robot to a final concentration of 10 μM with the final DMSO in each well at 0.1%. DMSO alone at this concentration had no effect on culture growth. The multi-well plates were sealed using Breath-easy Sealing membranes (sigma Z380059). The OD_600_ of each plate was read every 30 min using a Thermo Multiskan attached to an S&Banks P robotic plate handler. Data processing was achieved using R statistical software. To measure growth, we calculated the area under the curve (AUC) using the trapezoidal rule.

### Laurdan assay for membrane fluidity

The assay was modified from that of Gohrbandt (2022). Cultures were grown in NB/MSM at 30^°^C to exponential phase (OD 0.2) then washed with PBS:1M Na-Succinate (1:1) supplemented with 0.1% glucose, then were resuspended in the same buffer. Laurdan dye (6-dodecanoyl-2–dimethylaminonaphthalene) was added to a final concentration of 10 μM and the mixture was incubated for 10 min at 30^°^C in the dark with gentle shaking. The cell suspension was then aliquoted into 2 mL microcentrifuge tubes and centrifuged for 2 min at 5,500 rpm in an Eppendorf bench centrifuge. The cells were washed using the same buffer four times to remove the excess dye, and finally resuspended to an OD600_nm_ of 0.5 in PBS/succinate buffer before adding to a pre warmed black 96 well plate. As a positive control, the cells were incubated with 50 mM of the membrane fluidizer, benzyl alcohol. The cells were distributed into 96 well plates in the dark. After 10 min incubation at 30^°^C in a BMG plate reader, test and control compounds were added to give a total volume of 150 μL/well. The OD of the drug-treated or untreated cultures was measured at 10 min intervals. The Laurdan GP value was calculated using the equation: GP = (I_435nm_–I_490nm_)/(I_435nm_+I_490nm_). Reduced Laurdan GP value means increased fluidity.

### Measuring effects on a membrane-fluidity-sensitized *B. subtilis* strain

The cells were grown overnight in a minimal medium containing 1% (w/v) glucose, 50 μg per ml L-tryptophan, 0.2% (w/v) casein hydrolysate, MnS0_4_.4H_2_O (0.5 μM) and CaCl_2_ (50 μM). The medium was then supplemented with 100 μM of fatty-acid synthesis precursors, 2-methylbutyric acid (MB) or isobutyric acid (IB). The cells then were diluted to an OD600_nm_ of 0.02 in pre-warmed medium before being distributed in a pre-warmed 96 well plate. The cultures were incubated in a BMG SPECTROstar plate reader under shaking conditions for 1 h then manidipine, flunarizine or zoledronic acid (syn: Zoledronate) were added to the final concentration of 25–40 μM. The OD600_nm_ measurements were achieved ever 10 min for 16 h. All steps of the experiment was performed at 30^°^C.

The doubling time (the slope of the growth curve) was calculated for linear growth curve with Y axis logarithmic scale, using the exponential curve fitting equation, *y = Ae*^Bx^** and then, *Td = ln_2_/B*. As ln1 = 0, the equation was simplified to: *Td = ln2/B* = *0.693/B*. *B* value was specific to each growth line, generated by Excel.

### Microscopic imaging

Overnight cultures, grown in MSM at 30^°^C, were diluted (1:100 v/v) into fresh medium and grown at 30^°^C until exponential phase. The exponentially growing cultures were then diluted again to an OD of 0.02, and distributed into 24 wells plates, 1 mL/well. Manidipine, flunarizine, and zoledronic acid were added to the final concentration of 25, 12 μM, respectively. For the “no compound” control, DMSO was added to a final concentration of 0.025 or 0.010%. Growth of the cultures was continued at 30^°^C. For microscopy, a 2 μl sample of cell culture was mounted onto a microscope slide. Images were acquired with a Rolera EM-C2 (Q-imaging) camera attached to a Nikon Ti microscope using METAMORPH version 6 (Molecular Devices). Images were processed using Fiji/ImageJ; https://imagej.net/Fiji, (Schindelin et al., 2012).

### Liquid assay using MSM medium and meclizine in multi-well plates

MSM-NB was inoculated with one single colony (walled cells) or a 1:100 dilution of a 24 h culture (L-form) and incubated at 30^°^C overnight. The next morning the overnight culture was diluted in fresh MSM-NB and was grown to exponential phase. Each culture was diluted again in the same medium to an OD 600 nm of 0.02, then aliquoted into 96-well plates. Cultures were grown for 1 h and then challenged with a dilution series of meclizine (made in MSM-NB) to give a final volume of 200 μL. Culture growth was monitored for 12 h at 30^°^C, using a FluroStar (BMG LABTECH) plate reader.

## Data availability statement

The original contributions presented in this study are included in the article/Supplementary material, further inquiries can be directed to the corresponding author.

## Author contributions

KE, LW, and PB did the experiments. JE wrote the main text with contributions from all co-authors. All authors contributed to experimental design and concepts and analysed the data.

## Footnotes

1
https://www.selleckchem.com/screening/fda-approved-drug-library.html

## Supplementary material

Supplementary Figure 1 Schematic representation of the chromosomal region deleted in strain 18. The relative position of the deleted 18 Kbp compared to *B. subtilis* reference genome (AL009126, https://www.ncbi.nlm.nih.gov/genbank/) is shown. The data was generated using Qiagen CLC Bio software.

Supplementary Figure 2 Test for recovery of L-form growth after treatment with specific inhibitors. L-form strain Δ18 was grown in MSM/NB medium for 22 h at 30^°^C, then the culture was diluted and aliquots of the culture were treated with 5 or 20 μM of manidipine (light green and dark green, respectively) or flunarizine (light and dark blue), or a similar volume (0.5 μL) of DMSO only (black), to give a total volume of 200 μL in the wells of a 96 well plate (rows A to D). Growth (OD_600_) in each well (dotted lines) was recorded continuously. After 22 h a 10 μL sample of each culture was diluted into 190 μL pre warmed fresh medium with no compound addition (rows E to H). Culture growth was then continued for a further 20 h. Colors are as above but with solid lines. Note that the solid line values prior to dilution show the background levels of absorbance for empty wells. Each row contained three replicates and plots shown are averages of the three.

Supplementary Video 1 Time-lapse movie of *B. subtilis* L-forms (strain Δ18) after adding DMSO to 1% (left) or manidipine (25 μM; right). The cells were grown in a CELLview culture dish (Greiner Bio-One Ltd., UK) at 32^°^C. The 1st frame was taken 30 min after the addition of the compounds. 3 min per frame.
