# Supplementary material for: Repurposing drugs with specific activity against L-form bacteria
Source: Front Microbiol. 2023 Apr 4;14:1097413. doi: 10.3389/fmicb.2023.1097413 (PMC10110866; doi:10.3389/fmicb.2023.1097413)
Supplement: Supplementary file 1 [file Table_1.docx]

**Supplementary Table S1. First screen of the FDA compound set.**

| **Common Name** | **AUC L-forms** | **AUC Walled**  **cells** | **Residuals** |
| --- | --- | --- | --- |
| TROXIPIDE | 0.0047 | 0.2284 | -0.1758 |
| EXALAMIDE | 0.0040 | 0.2195 | -0.1694 |
| BAMBUTEROL HYDROCHLORIDE | 0.0080 | 0.2141 | -0.1612 |
| BENPROPERINE PHOSPHATE | 0.0081 | 0.2080 | -0.1563 |
| CARPROFEN | 0.0064 | 0.1948 | -0.1475 |
| MANIDIPINE (MANYPER) | 0.0093 | 0.1904 | -0.1411 |
| IVERMECTIN | 0.0071 | 0.1829 | -0.1374 |
| ELVITEGRAVIR (GS-9137) | 0.0099 | 0.1840 | -0.1355 |
| MECLIZINE 2HCL | 0.0089 | 0.1810 | -0.1341 |
| FLUNARIZINE 2HCL | 0.0058 | 0.1752 | -0.1326 |
| APREPITANT (MK-0869) | 0.0110 | 0.1763 | -0.1283 |
| AMG-073 HCL (CINACALCET HYDROCHLORIDE) | 0.0079 | 0.1713 | -0.1274 |
| TRETINOIN (ABERELA) | 0.0070 | 0.1686 | -0.1262 |
| TRIFLUOPERAZINE 2HCL | 0.0195 | 0.1834 | -0.1254 |
| BORTEZOMIB (VELCADE) | 0.0048 | 0.1641 | -0.1249 |
| SECNIDAZOLE | 0.0411 | 0.2064 | -0.1220 |
| ETRAVIRINE (TMC125) | 0.0113 | 0.1659 | -0.1197 |
| TAMOXIFEN CITRATE (NOLVADEX) | 0.0045 | 0.1543 | -0.1173 |
| FOSAPREPITANT DIMEGLUMINE | 0.0100 | 0.1596 | -0.1161 |
| DRONEDARONE HCL (MULTAQ) | 0.0069 | 0.1418 | -0.1052 |
| TEMSIROLIMUS (TORISEL) | 0.0307 | 0.1706 | -0.1041 |
| NICLOSAMIDE (NICLOCIDE) | 0.0111 | 0.1342 | -0.0949 |
| ACITRETIN | 0.0284 | 0.1507 | -0.0906 |
| BLEOMYCIN SULFATE | 0.0086 | 0.1253 | -0.0904 |
| NITROFURAZONE (NITROFURAL) | 0.0298 | 0.1505 | -0.0891 |
| AMIODARONE HCL | 0.0104 | 0.1178 | -0.0827 |
| PHENPROBAMATE | 0.0986 | 0.2254 | -0.0795 |
| CILNIDIPINE | 0.0757 | 0.1919 | -0.0759 |
| ANAGRELIDE HCL | 0.0807 | 0.1972 | -0.0751 |
| RAPAMYCIN (SIROLIMUS) | 0.0964 | 0.2144 | -0.0730 |
| TERBINAFINE (LAMISIL, TERBINEX) | 0.0753 | 0.1873 | -0.0727 |
| FLUNISOLIDE | 0.0875 | 0.2012 | -0.0714 |
| DICLOFENAC POTASSIUM | 0.0671 | 0.1707 | -0.0677 |
| ZOLEDRONATE | 0.0938 | 0.2002 | -0.0644 |
| NIFLUMIC ACID | 0.0413 | 0.1331 | -0.0638 |
| TOREMIFENE CITRATE (FARESTON, ACAPODENE) | 0.0448 | 0.1351 | -0.0620 |
| APRACLONIDINE HCL | 0.1013 | 0.2056 | -0.0611 |
| TIOCONAZOLE | 0.0067 | 0.0857 | -0.0610 |
| TOBRAMYCIN | 0.0084 | 0.0859 | -0.0595 |
| LOPINAVIR (ABT-378) | 0.0809 | 0.1771 | -0.0590 |
| BUTENAFINE HCL | 0.1095 | 0.2110 | -0.0572 |
| SIMVASTATIN (ZOCOR) | 0.0721 | 0.1631 | -0.0568 |
| ZAFIRLUKAST (ACCOLATE) | 0.0079 | 0.0819 | -0.0568 |
| QUETIAPINE FUMARATE (SEROQUEL) | 0.1444 | 0.2518 | -0.0545 |
| ISRADIPINE (DYNACIRC) | 0.1343 | 0.2362 | -0.0523 |
| LIRANAFTATE | 0.0937 | 0.1841 | -0.0518 |
| DOXIFLURIDINE | 0.0819 | 0.1655 | -0.0489 |
| BUFLOMEDIL HYDROCHLORIDE | 0.1193 | 0.2124 | -0.0485 |
| TADALAFIL (CIALIS) | 0.1405 | 0.2374 | -0.0471 |
| EZETIMIBE (ZETIA) | 0.0848 | 0.1661 | -0.0464 |
| DOCOSANOL (ABREVA) | 0.1445 | 0.2411 | -0.0459 |
| DICLAZURIL | 0.0151 | 0.0769 | -0.0456 |
| UNKNOWN | 0.0065 | 0.0649 | -0.0448 |
| TENOFOUIR | 0.1259 | 0.2116 | -0.0413 |
| CLOFIBRATE (ATROMID-S) | 0.0954 | 0.1720 | -0.0405 |
| RIFABUTIN (MYCOBUTIN) | 0.0137 | 0.0661 | -0.0385 |
| OFLOXACIN (FLOXIN) | 0.0890 | 0.1598 | -0.0372 |
| FELODIPINE (PLENDIL) | 0.0862 | 0.1552 | -0.0364 |
| ADRUCIL (FLUOROURACIL) | 0.0727 | 0.1379 | -0.0362 |
| MITOXANTRONE HYDROCHLORIDE | 0.1098 | 0.1847 | -0.0361 |
| HYGROMYCIN B | 0.1102 | 0.1850 | -0.0359 |
| L-THYROXINE | 0.1093 | 0.1833 | -0.0356 |
| MARBOFLOXACIN | 0.0901 | 0.1589 | -0.0354 |
| MASITINIB (AB1010) | 0.1209 | 0.1961 | -0.0341 |
| SULFADOXINE | 0.1350 | 0.2130 | -0.0332 |
| FENOFIBRATE (TRICOR, TRILIPIX) | 0.1161 | 0.1881 | -0.0325 |
| VALPROIC ACID SODIUM SALT (SODIUM VALPROATE) | 0.1653 | 0.2500 | -0.0322 |
| UNKNOWN | 0.1056 | 0.1726 | -0.0307 |
| NITAZOXANIDE (ALINIA, ANNITA) | 0.0153 | 0.0581 | -0.0305 |
| THIAMPHENICOL (THIOPHENICOL) | 0.0232 | 0.0679 | -0.0304 |
| ATORVASTATIN CALCIUM (LIPITOR) | 0.1184 | 0.1881 | -0.0302 |
| FUDOSTEINE | 0.1236 | 0.1941 | -0.0298 |
| FENTICONAZOLE NITRATE | 0.0060 | 0.0451 | -0.0296 |
| AMPHOTERICIN B (ABELCET) | 0.1653 | 0.2466 | -0.0295 |
| MIGLITOL (GLYSET) | 0.1483 | 0.2243 | -0.0289 |
| AMLEXANOX | 0.1342 | 0.2061 | -0.0287 |
| AMOROLFINE HYDROCHLORIDE | 0.1188 | 0.1866 | -0.0286 |
| FLUVASTATIN SODIUM (LESCOL) | 0.1070 | 0.1715 | -0.0285 |
| TIMOLOLMALEATE | 0.1468 | 0.2217 | -0.0284 |
| ETOPOSIDE (VP-16) | 0.1167 | 0.1835 | -0.0283 |
| BENAZEPRIL | 0.1332 | 0.2028 | -0.0270 |
| TENATOPRAZOLE | 0.1564 | 0.2321 | -0.0270 |
| GEMCITABINE HCL (GEMZAR) | 0.0202 | 0.0590 | -0.0264 |
| PIMECROLIMUS | 0.1170 | 0.1808 | -0.0259 |
| RIBOSTAMYCIN SULPHATE | 0.1430 | 0.2137 | -0.0258 |
| RUBETICAN | 0.1529 | 0.2258 | -0.0255 |
| STREPRONIN | 0.1522 | 0.2247 | -0.0253 |
| TIBOLONE | 0.1520 | 0.2243 | -0.0252 |
| DULOXETINE HCL (CYMBALTA) | 0.1268 | 0.1921 | -0.0250 |
| CEFIXIME | 0.0040 | 0.0361 | -0.0245 |
| ETHINYL ESTRADIOL | 0.1186 | 0.1811 | -0.0245 |
| ENOXACIN | 0.1287 | 0.1935 | -0.0241 |
| SULINDAC (CLINORIL) | 0.1231 | 0.1862 | -0.0240 |
| NITRENDIPINE | 0.1349 | 0.2011 | -0.0240 |
| RESERPINE | 0.1155 | 0.1765 | -0.0239 |
| PIDOTIMOD | 0.1477 | 0.2173 | -0.0239 |
| LIDOCAINE (ALPHACAINE) | 0.1325 | 0.1980 | -0.0239 |
| NIACIN (NICOTINIC ACID) | 0.1520 | 0.2226 | -0.0239 |
| FK-506 (TACROLIMUS) | 0.1098 | 0.1688 | -0.0236 |
| TENIPOSIDE (VUMON) | 0.0434 | 0.0845 | -0.0233 |
| FULVESTRANT (FASLODEX) | 0.1208 | 0.1821 | -0.0230 |
| NAD+ | 0.1498 | 0.2186 | -0.0229 |
| DICLOFENAC | 0.1040 | 0.1605 | -0.0228 |
| NADIFLOXACIN | 0.0049 | 0.0346 | -0.0224 |
| VARDENAFIL CITRATE | 0.1411 | 0.2067 | -0.0221 |
| PACLITAXEL (TAXOL) | 0.1442 | 0.2105 | -0.0221 |
| BIBR-1048 (DABIGATRAN) | 0.1458 | 0.2123 | -0.0219 |
| ACTARIT | 0.1439 | 0.2093 | -0.0214 |
| MECARBINATE | 0.1312 | 0.1928 | -0.0211 |
| TOLTERODINE TARTRATE | 0.1577 | 0.2260 | -0.0208 |
| RILUZOLE (RILUTEK) | 0.1409 | 0.2047 | -0.0208 |
| DIETHYLSTILBESTROL (STILBESTROL) | 0.1016 | 0.1548 | -0.0207 |
| CELECOXIB | 0.1252 | 0.1846 | -0.0206 |
| BETAMETHASONE (CELESTONE) | 0.1419 | 0.2053 | -0.0203 |
| PROPAFENONE (RYTMONORM) | 0.1409 | 0.2040 | -0.0202 |
| INDACATEROL MALEATE | 0.1233 | 0.1814 | -0.0200 |
| NAFAMOSTAT MESYLATE | 0.1189 | 0.1759 | -0.0200 |
| SPECTINOMYCIN HYDROCHLORIDE | 0.1230 | 0.1802 | -0.0193 |
| SULFAPYRIDINE (DAGENAN) | 0.1351 | 0.1949 | -0.0189 |
| TRIMEBUTINE MALEATE | 0.1536 | 0.2178 | -0.0185 |
| TOLFENAMIC ACID | 0.1081 | 0.1601 | -0.0185 |
| FOSINOPRIL NA | 0.1654 | 0.2326 | -0.0183 |
| CLOZAPINE (CLOZARIL) | 0.1210 | 0.1764 | -0.0183 |
| IVABRADINE HCL (PROCORALAN) | 0.1336 | 0.1923 | -0.0183 |
| RITONAVIR | 0.1356 | 0.1941 | -0.0177 |
| LAFUTIDINE | 0.1412 | 0.2012 | -0.0177 |
| SULPIRIDE | 0.1615 | 0.2269 | -0.0177 |
| AMIKACIN SULPHATE | 0.1232 | 0.1783 | -0.0177 |
| CLENBUTEROL HCL | 0.1501 | 0.2124 | -0.0176 |
| FORMESTANE | 0.1630 | 0.2285 | -0.0176 |
| CREATININE | 0.1434 | 0.2034 | -0.0173 |
| TICLOPIDINE HCL | 0.1559 | 0.2191 | -0.0172 |
| SELEGILINE | 0.1590 | 0.2227 | -0.0169 |
| CLONAZEPAM | 0.1575 | 0.2206 | -0.0168 |
| BIBR 953 (DABIGATRAN ETEXILATE, PRADAXA) | 0.1483 | 0.2084 | -0.0163 |
| PHENYLEPHRINE HCL | 0.1412 | 0.1992 | -0.0162 |
| ARBIDOL HCL | 0.1280 | 0.1825 | -0.0162 |
| BROMHEXINE HCL | 0.1418 | 0.1993 | -0.0157 |
| NAFTIFINE HCL | 0.1358 | 0.1916 | -0.0156 |
| CONIVAPTAN HCL (VAPRISOL) | 0.1271 | 0.1805 | -0.0156 |
| PHENINDIONE (RECTADIONE) | 0.1374 | 0.1933 | -0.0154 |
| OMEPRAZOLE (PRILOSEC) | 0.1393 | 0.1954 | -0.0151 |
| SAXAGLIPTIN (BMS-477118,ONGLYZA) | 0.1419 | 0.1987 | -0.0151 |
| ENTECAVIR HYDRATE | 0.1444 | 0.2019 | -0.0150 |
| FUROSEMIDE (LASIX) | 0.1431 | 0.2002 | -0.0150 |
| ETODOLAC (LODINE) | 0.1231 | 0.1745 | -0.0148 |
| NAPROXEN (ALEVE) | 0.1269 | 0.1793 | -0.0148 |
| PRALATREXATE (FOLOTYN) | 0.1392 | 0.1948 | -0.0147 |
| ACEMETACIN (EMFLEX) | 0.1352 | 0.1895 | -0.0145 |
| POSACONAZOLE | 0.1479 | 0.2054 | -0.0144 |
| CLEBOPRIDE MALEATE | 0.1464 | 0.2033 | -0.0142 |
| SULFASALAZINE | 0.1601 | 0.2205 | -0.0141 |
| LEFLUNOMIDE | 0.1335 | 0.1867 | -0.0140 |
| ENROFLOXACIN | 0.0444 | 0.0736 | -0.0137 |
| ANASTROZOLE | 0.1543 | 0.2125 | -0.0136 |
| PRAMIPEXOLE DIHYDROCHLORIDE MONOHYDRATE | 0.1648 | 0.2258 | -0.0135 |
| INDAPAMIDE (LOZOL) | 0.1353 | 0.1883 | -0.0135 |
| ESTRIOL | 0.1647 | 0.2253 | -0.0133 |
| DARIFENACIN HBR | 0.1322 | 0.1840 | -0.0132 |
| LOPERAMIDE HYDROCHLORIDE | 0.1262 | 0.1762 | -0.0130 |
| DOFETILIDE (TIKOSYN) | 0.1412 | 0.1946 | -0.0126 |
| THIAZMAZOLE | 0.1510 | 0.2070 | -0.0125 |
| MINOXIDIL | 0.1427 | 0.1961 | -0.0122 |
| NEBIVOLOL (BYSTOLIC) | 0.1326 | 0.1831 | -0.0121 |
| CISPLATIN | 0.1378 | 0.1897 | -0.0120 |
| FEPRAZONE | 0.1523 | 0.2079 | -0.0120 |
| ARTEMETHER (SM-224) | 0.1408 | 0.1931 | -0.0117 |
| ADEFOVIR DIPIVOXIL (PREVEON, HEPSERA) | 0.1412 | 0.1934 | -0.0116 |
| PROTIONAMIDE (PROTHIONAMIDE) | 0.1409 | 0.1927 | -0.0113 |
| CETIRIZINE DIHYDROCHLORIDE | 0.1547 | 0.2100 | -0.0112 |
| TETRACYCLINE HCL | 0.0809 | 0.1164 | -0.0110 |
| NALTREXONE HCL | 0.1494 | 0.2030 | -0.0110 |
| AMINOGLUTETHIMIDE | 0.1640 | 0.2214 | -0.0109 |
| DAPOXETINE HYDROCHLORIDE (PRILIGY) | 0.1374 | 0.1876 | -0.0108 |
| CLEVIPREX (CLEVIDIPINE) | 0.1411 | 0.1921 | -0.0106 |
| CLOTRIMAZOLE (CANESTEN) | 0.0042 | 0.0185 | -0.0104 |
| GLIQUIDONE | 0.1056 | 0.1468 | -0.0104 |
| BIAPENEM | 0.1507 | 0.2035 | -0.0101 |
| BROMPHENIRAMINE | 0.1401 | 0.1900 | -0.0100 |
| SULFADOXINE (SULPHADOXINE) | 0.1428 | 0.1932 | -0.0098 |
| PHENACETIN | 0.1446 | 0.1953 | -0.0098 |
| LONIDAMINE | 0.1178 | 0.1615 | -0.0097 |
| PYRIDOSTIGMINE BROMIDE (MESTINON) | 0.1439 | 0.1943 | -0.0095 |
| TRILOSTANE | 0.1333 | 0.1807 | -0.0095 |
| PHENOXYBENZAMINE HCL | 0.1548 | 0.2078 | -0.0094 |
| NEVIRAPINE (VIRAMUNE) | 0.1497 | 0.2012 | -0.0093 |
| ARIPIPRAZOLE (ABILIFY) | 0.1350 | 0.1825 | -0.0092 |
| ESOMEPRAZOLE MAGNESIUM (NEXIUM) | 0.1380 | 0.1863 | -0.0092 |
| PALIPERIDONE (INVEGA) | 0.1408 | 0.1896 | -0.0090 |
| ORLISTAT (ALLI, XENICAL) | 0.1423 | 0.1915 | -0.0090 |
| ALTRENOGEST | 0.1438 | 0.1933 | -0.0089 |
| STANOZOLOL | 0.1496 | 0.2006 | -0.0089 |
| PIZOTIFEN | 0.1596 | 0.2130 | -0.0087 |
| MOEXIPRIL HCL | 0.1475 | 0.1974 | -0.0084 |
| CLOPIDOGREL (PLAVIX) | 0.1290 | 0.1739 | -0.0083 |
| STREPTOMYCIN SULFATE | 0.1305 | 0.1756 | -0.0083 |
| MIANSERIN HYDROCHLORIDE | 0.1302 | 0.1752 | -0.0082 |
| DOXERCALCIFEROL (HECTOROL) | 0.1395 | 0.1869 | -0.0081 |
| BICALUTAMIDE (CASODEX) | 0.1386 | 0.1855 | -0.0079 |
| EXEMESTANE | 0.1645 | 0.2181 | -0.0078 |
| KETOTIFEN FUMARATE (ZADITOR) | 0.1441 | 0.1922 | -0.0077 |
| PRIMIDONE (MYSOLINE) | 0.1371 | 0.1832 | -0.0077 |
| EPALRESTAT | 0.1340 | 0.1792 | -0.0076 |
| SCOPINE | 0.1452 | 0.1933 | -0.0075 |
| ETORICOXIB | 0.1659 | 0.2195 | -0.0075 |
| METOLAZONE (ZAROXOLYN) | 0.1595 | 0.2113 | -0.0074 |
| ALFUZOSIN HYDROCHLORIDE (UROXATRAL) | 0.1301 | 0.1740 | -0.0073 |
| DIMETHYL FUMARATE | 0.1418 | 0.1887 | -0.0073 |
| ALENDRONATE (FOSAMAX) | 0.1432 | 0.1905 | -0.0073 |
| TRANILAST (SB 252218) | 0.1301 | 0.1736 | -0.0071 |
| ADIPHENINE HCL | 0.1456 | 0.1932 | -0.0070 |
| CYCLOSPORINE (NEORAL) | 0.1366 | 0.1816 | -0.0069 |
| CIDOFOVIR (VISTIDE) | 0.1386 | 0.1841 | -0.0069 |
| XYLOMETAZOLINE HCL | 0.1404 | 0.1860 | -0.0065 |
| TRIMEBUTINE | 0.1394 | 0.1846 | -0.0065 |
| DOMPERIDONE (MOTILIUM) | 0.1393 | 0.1844 | -0.0064 |
| EVISTA (RALOXIFENE HYDROCHLORIDE) | 0.1338 | 0.1774 | -0.0063 |
| SULFAMETHAZINE | 0.1558 | 0.2051 | -0.0062 |
| CLOBETASOL PROPIONATE | 0.1407 | 0.1859 | -0.0062 |
| TIOPRONIN (THIOLA) | 0.1746 | 0.2288 | -0.0062 |
| SITAFLOXACIN HYDRATE | 0.0095 | 0.0197 | -0.0061 |
| NICOTINAMIDE (NIACINAMIDE) | 0.1413 | 0.1863 | -0.0059 |
| AMITRIPTYLINE HCL | 0.1406 | 0.1854 | -0.0058 |
| (+,-)-OCTOPAMINE HCL | 0.1500 | 0.1971 | -0.0058 |
| MIZOLASTINE (MIZOLLEN) | 0.1467 | 0.1930 | -0.0058 |
| CILAZAPRIL MONOHYDRATE (INHIBACE) | 0.1473 | 0.1936 | -0.0057 |
| NATAMYCIN (PIMARICIN) | 0.1310 | 0.1729 | -0.0056 |
| MITIGLINIDE CALCIUM | 0.1356 | 0.1786 | -0.0055 |
| ANTIPYRINE | 0.1460 | 0.1917 | -0.0054 |
| GLYBURIDE (DIABETA) | 0.1249 | 0.1649 | -0.0054 |
| MENADIONE | 0.1296 | 0.1708 | -0.0054 |
| AZASETRON HCL (Y-25130) | 0.1452 | 0.1905 | -0.0053 |
| FLUOXETINE HCL | 0.1342 | 0.1765 | -0.0053 |
| ARTEMISININ | 0.1486 | 0.1947 | -0.0053 |
| MIRTAZAPINE (REMERON, AVANZA) | 0.1352 | 0.1778 | -0.0053 |
| CHLORPROTHIXENE | 0.1410 | 0.1852 | -0.0052 |
| MEDROXYPROGESTERONE ACETATE | 0.1444 | 0.1894 | -0.0052 |
| TEMOCAPRIL HCL | 0.1444 | 0.1893 | -0.0052 |
| ERLOTINIB HCL | 0.1529 | 0.1998 | -0.0050 |
| DOXORUBICIN (ADRIAMYCIN) | 0.0102 | 0.0191 | -0.0049 |
| FLUOCINOLONE ACETONIDE (FLUCORT-N) | 0.1565 | 0.2043 | -0.0048 |
| NIMODIPINE (NIMOTOP) | 0.1520 | 0.1982 | -0.0046 |
| ADENOSINE (ADENOCARD) | 0.1583 | 0.2062 | -0.0045 |
| IAMRINONE | 0.1561 | 0.2033 | -0.0045 |
| AMFEBUTAMONE (BUPROPION) | 0.1453 | 0.1893 | -0.0043 |
| METHYLDOPA (ALDOMET) | 0.1639 | 0.2127 | -0.0042 |
| ILOPERIDONE (FANAPT) | 0.1317 | 0.1720 | -0.0041 |
| GEFITINIB (IRESSA) | 0.1400 | 0.1824 | -0.0041 |
| NAFTOPIDIL (FLIVAS) | 0.1488 | 0.1935 | -0.0041 |
| TRICHLORMETHIAZIDE (ACHLETIN) | 0.1332 | 0.1737 | -0.0040 |
| TAZOBACTAM NA | 0.1610 | 0.2088 | -0.0040 |
| GABEXATE MESYLATE | 0.1491 | 0.1938 | -0.0040 |
| LEVODOPA (SINEMET) | 0.1384 | 0.1801 | -0.0038 |
| RIBAVIRIN (COPEGUS) | 0.1386 | 0.1802 | -0.0037 |
| IDEBENONE | 0.1494 | 0.1938 | -0.0037 |
| CALCITRIOL (ROCALTROL) | 0.1303 | 0.1697 | -0.0037 |
| TENOFOVIR DISOPROXIL FUMARATE | 0.1533 | 0.1986 | -0.0036 |
| DYCLONINE HCL | 0.1499 | 0.1943 | -0.0035 |
| BETAMETHASONE VALERATE (BETNOVATE) | 0.1580 | 0.2044 | -0.0035 |
| ROFLUMILAST (DAXAS) | 0.1428 | 0.1850 | -0.0034 |
| MEGESTROL ACETATE | 0.1373 | 0.1779 | -0.0032 |
| CHENODEOXYCHOLIC ACID | 0.1435 | 0.1857 | -0.0032 |
| PRAVASTATIN SODIUM | 0.1432 | 0.1853 | -0.0032 |
| 10-DAB (10-DEACETYLBACCATIN) | 0.1465 | 0.1895 | -0.0032 |
| PANTOTHENIC ACID (PANTOTHENATE) | 0.1441 | 0.1862 | -0.0030 |
| SULBACTAM | 0.1365 | 0.1766 | -0.0030 |
| PIOGLITAZONE HCL (ACTOS) | 0.1385 | 0.1789 | -0.0029 |
| METHSCOPOLAMINE (PAMINE) | 0.1391 | 0.1795 | -0.0027 |
| STAVUDINE | 0.1464 | 0.1887 | -0.0027 |
| DIENOGEST | 0.1503 | 0.1934 | -0.0025 |
| LISINOPRIL (ZESTRIL) | 0.1525 | 0.1962 | -0.0025 |
| BENZTROPINE MESYLATE | 0.1460 | 0.1880 | -0.0025 |
| TRIPELENNAMINE HCL | 0.1352 | 0.1743 | -0.0025 |
| ADENINE | 0.1380 | 0.1777 | -0.0024 |
| LINCOMYCIN HYDROCHLORIDE (LINCOCIN) | 0.0910 | 0.1180 | -0.0022 |
| CAPTOPRIL (CAPOTEN) | 0.1431 | 0.1839 | -0.0021 |
| DUTASTERIDE | 0.1418 | 0.1821 | -0.0020 |
| SULFAMETHIZOLE (PROKLAR) | 0.1388 | 0.1782 | -0.0020 |
| MOXONIDINE | 0.1412 | 0.1811 | -0.0019 |
| DEXRAZOXANE HYDROCHLORIDE | 0.1571 | 0.2012 | -0.0018 |
| OXYBUTYNIN (DITROPAN) | 0.1470 | 0.1884 | -0.0018 |
| (R)-BACLOFEN | 0.1424 | 0.1820 | -0.0013 |
| LEVETIRACETAM | 0.1464 | 0.1868 | -0.0011 |
| QUINAPRIL HCL (ACCUPRIL) | 0.1436 | 0.1832 | -0.0011 |
| DIDANOSINE (VIDEX) | 0.1537 | 0.1958 | -0.0010 |
| RIVASTIGMINE TARTRATE (EXELON) | 0.1550 | 0.1975 | -0.0010 |
| LAPATINIB DITOSYLATE (TYKERB) | 0.1377 | 0.1756 | -0.0010 |
| PAEONIFLORIN | 0.1465 | 0.1867 | -0.0010 |
| ARGATROBAN | 0.1358 | 0.1731 | -0.0009 |
| FLOXURIDINE | 0.0057 | 0.0084 | -0.0009 |
| LEVONORGESTREL (LEVONELLE) | 0.1373 | 0.1750 | -0.0009 |
| CYPROHEPTADINE HCL (PERIACTIN) | 0.1598 | 0.2033 | -0.0008 |
| D-CYCLOSERINE | 0.1364 | 0.1737 | -0.0008 |
| FLUNIXIN MEGLUMIN | 0.1343 | 0.1706 | -0.0005 |
| ETOMIDATE | 0.1348 | 0.1712 | -0.0005 |
| SILDENAFIL CITRATE | 0.1527 | 0.1939 | -0.0004 |
| REBAMIPIDE | 0.1356 | 0.1721 | -0.0004 |
| PRILOCAINE | 0.1449 | 0.1839 | -0.0003 |
| WARFARIN | 0.1425 | 0.1807 | -0.0003 |
| PERINDOPRIL ERBUMINE (ACEON) | 0.1471 | 0.1864 | -0.0002 |
| DANOFLOXACIN MESYLATE | 0.0071 | 0.0091 | -0.0001 |
| DETOMIDINE HCL | 0.1475 | 0.1868 | -0.0001 |
| DEXAMETHASONE ACETATE | 0.1589 | 0.2012 | 0.0000 |
| AMLODIPINE BESYLATE (NORVASC) | 0.1478 | 0.1869 | 0.0001 |
| LEUCOVORIN CALCIUM | 0.1471 | 0.1860 | 0.0002 |
| OLMESARTAN MEDOXOMIL (BENICAR) | 0.1478 | 0.1866 | 0.0004 |
| SILODOSIN (RAPAFLO) | 0.1335 | 0.1684 | 0.0005 |
| CEPHALOMANNINE | 0.1555 | 0.1961 | 0.0006 |
| DISODIUM CROMOGLYCATE | 0.1572 | 0.1982 | 0.0006 |
| FEXOFENADINE HCL | 0.1321 | 0.1661 | 0.0008 |
| OXYTETRACYCLINE (TERRAMYCIN) | 0.1368 | 0.1721 | 0.0008 |
| GABAPENTIN (NEURONTIN) | 0.1631 | 0.2053 | 0.0009 |
| FLUDARABINE (FLUDARA) | 0.1245 | 0.1564 | 0.0009 |
| RISPERIDONE (RISPERDAL) | 0.1419 | 0.1784 | 0.0009 |
| SULFAMETHOXAZOLE | 0.1440 | 0.1810 | 0.0010 |
| FELBAMATE | 0.1547 | 0.1940 | 0.0014 |
| ACETYLCYSTEINE | 0.1444 | 0.1810 | 0.0014 |
| VITAMIN C (ASCORBIC ACID) | 0.1970 | 0.2474 | 0.0016 |
| OXYMETAZOLINE HYDROCHLORIDE | 0.1401 | 0.1753 | 0.0017 |
| SPIRAMYCIN | 0.0033 | 0.0020 | 0.0017 |
| PHENYLBUTAZONE (BUTAZOLIDIN, BUTATRON) | 0.1375 | 0.1718 | 0.0018 |
| ABACAVIR SULFATE | 0.1484 | 0.1856 | 0.0018 |
| PIZOTIFEN MALATE | 0.1703 | 0.2133 | 0.0018 |
| TENOFOVIR (VIREAD) | 0.1503 | 0.1878 | 0.0019 |
| PIPERACILLIN | 0.1453 | 0.1814 | 0.0020 |
| ARECOLINE | 0.1363 | 0.1698 | 0.0022 |
| ERYTHROMYCIN (E-MYCIN) | 0.0053 | 0.0039 | 0.0022 |
| DL-ADRENALINE | 0.1471 | 0.1831 | 0.0024 |
| TELMISARTAN (MICARDIS) | 0.1379 | 0.1715 | 0.0024 |
| TELBIVUDINE (SEBIVO, TYZEKA) | 0.1433 | 0.1783 | 0.0024 |
| PREGNENOLONE | 0.1546 | 0.1927 | 0.0024 |
| GATIFLOXACIN | 0.0055 | 0.0039 | 0.0025 |
| TRIAMCINOLONE (ARISTOCORT) | 0.1423 | 0.1769 | 0.0025 |
| AZACYCLONOL | 0.1454 | 0.1808 | 0.0025 |
| RIFAMPIN (RIFADIN, RIMACTANE) | 0.0059 | 0.0041 | 0.0027 |
| PYRIMETHAMINE | 0.1354 | 0.1678 | 0.0029 |
| MEPIVACAINE HCL | 0.1368 | 0.1693 | 0.0030 |
| L-ARGININE HCL | 0.1546 | 0.1916 | 0.0032 |
| CLONIDINE HYDROCHLORIDE (CATAPRES) | 0.1427 | 0.1766 | 0.0032 |
| STRONTIUM RANELATE (PROTELOS) | 0.1428 | 0.1767 | 0.0033 |
| NOVOBIOCIN SODIUM (ALBAMYCIN) | 0.0070 | 0.0046 | 0.0034 |
| FENOPROFEN CALCIUM HYDRATE | 0.1498 | 0.1854 | 0.0034 |
| TYLOSIN TARTRATE | 0.0056 | 0.0028 | 0.0034 |
| ATRACURIUM BESYLATE | 0.1475 | 0.1823 | 0.0035 |
| RIFAXIMIN (XIFAXAN) | 0.0068 | 0.0042 | 0.0035 |
| DOXAZOSIN MESYLATE | 0.1545 | 0.1911 | 0.0035 |
| RALTEGRAVIR (MK-0518) | 0.1531 | 0.1893 | 0.0036 |
| S-(+)-ROLIPRAM | 0.1505 | 0.1859 | 0.0037 |
| PREDNISONE (ADASONE) | 0.1579 | 0.1952 | 0.0037 |
| ROXITHROMYCIN (ROXL-150) | 0.0057 | 0.0025 | 0.0037 |
| VALDECOXIB | 0.1715 | 0.2124 | 0.0037 |
| CAMPTOTHECIN | 0.1547 | 0.1911 | 0.0037 |
| IBUPROFEN (ADVIL) | 0.1507 | 0.1860 | 0.0037 |
| CAPECITABINE (XELODA) | 0.1583 | 0.1955 | 0.0038 |
| BIOTIN (VITAMIN B7) | 0.1396 | 0.1718 | 0.0038 |
| XYLAZINE HCL | 0.1491 | 0.1838 | 0.0039 |
| NELARABINE (ARRANON) | 0.1771 | 0.2193 | 0.0039 |
| DORZOLAMIDE HCL | 0.1454 | 0.1791 | 0.0039 |
| METFORMIN HYDROCHLORIDE (GLUCOPHAGE) | 0.1455 | 0.1792 | 0.0039 |
| DYPHYLLINE (DILOR) | 0.1365 | 0.1677 | 0.0040 |
| QUININE HYDROCHLORIDE DIHYDRATE | 0.1688 | 0.2084 | 0.0042 |
| CLINDAMYCIN HYDROCHLORIDE (DALACIN) | 0.0079 | 0.0047 | 0.0042 |
| TIZANIDINE HCL | 0.1411 | 0.1730 | 0.0044 |
| LOTEPREDNOL ETABONATE | 0.1540 | 0.1893 | 0.0044 |
| CLOBAZAM | 0.1620 | 0.1994 | 0.0045 |
| FINASTERIDE | 0.1593 | 0.1959 | 0.0046 |
| ENALAPRILAT DIHYDRATE | 0.1381 | 0.1690 | 0.0046 |
| BUTOCONAZOLE NITRATE | 0.0058 | 0.0013 | 0.0048 |
| RAMIPRIL (ALTACE) | 0.1522 | 0.1866 | 0.0048 |
| ALLOPURINOL (ZYLOPRIM) | 0.1535 | 0.1883 | 0.0048 |
| FLEROXACIN | 0.1561 | 0.1915 | 0.0048 |
| BIPERIDEN HCL | 0.1552 | 0.1903 | 0.0049 |
| LINEZOLID (ZYVOX) | 0.0060 | 0.0013 | 0.0050 |
| DASATINIB (BMS-354825) | 0.1605 | 0.1968 | 0.0050 |
| ESTRONE | 0.1375 | 0.1676 | 0.0051 |
| UNKNOWN | 0.1474 | 0.1801 | 0.0052 |
| CILOSTAZOL | 0.1458 | 0.1779 | 0.0052 |
| CICLOPIROX (PENLAC) | 0.1429 | 0.1742 | 0.0053 |
| IDARUBICIN HCL | 0.0082 | 0.0037 | 0.0053 |
| NILOTINIB (AMN-107) | 0.1711 | 0.2098 | 0.0053 |
| SULBACTAN NA | 0.1758 | 0.2157 | 0.0054 |
| ENALAPRIL MALEATE (VASOTEC) | 0.1526 | 0.1862 | 0.0054 |
| NATEGLINIDE (STARLIX) | 0.1430 | 0.1741 | 0.0055 |
| RIZATRIPTAN BENZOATE (MAXALT) | 0.1457 | 0.1774 | 0.0056 |
| CHLORPROMAZINE (SONAZINE) | 0.1458 | 0.1775 | 0.0056 |
| DIRITHROMYCIN | 0.1523 | 0.1856 | 0.0056 |
| ALIBENDOL | 0.1462 | 0.1779 | 0.0057 |
| TORSEMIDE (DEMADEX) | 0.1408 | 0.1710 | 0.0057 |
| VORICONAZOLE | 0.1384 | 0.1679 | 0.0057 |
| CLOPRENALINE HCL | 0.1840 | 0.2257 | 0.0057 |
| ADAPALENE | 0.1481 | 0.1801 | 0.0058 |
| SALBUTAMOL SULFATE (ALBUTEROL) | 0.1530 | 0.1862 | 0.0059 |
| BETAPAR (MEPREDNISONE) | 0.1572 | 0.1914 | 0.0059 |
| PLERIXAFOR 8HCL (DB06809) | 0.1636 | 0.1995 | 0.0060 |
| URSODIOL (ACTIGAL URSO) | 0.1340 | 0.1620 | 0.0060 |
| ENTACAPONE | 0.1412 | 0.1710 | 0.0061 |
| MOXIFLOXACIN HYDROCHLORIDE | 0.0071 | 0.0013 | 0.0061 |
| EPIRUBICIN HYDROCHLORIDE | 0.0091 | 0.0038 | 0.0061 |
| GALLAMINE TRIETHIODIDE (FLAXEDIL) | 0.1449 | 0.1756 | 0.0061 |
| GENISTEIN | 0.1424 | 0.1724 | 0.0063 |
| PRAZIQUANTEL (BILTRICIDE) | 0.1704 | 0.2075 | 0.0065 |
| EMPTY | 0.1548 | 0.1878 | 0.0065 |
| TOLVAPTAN (OPC-41061) | 0.1603 | 0.1948 | 0.0065 |
| AVOBENZONE (PARSOL 1789) | 0.1435 | 0.1729 | 0.0069 |
| DAUNORUBICIN HCL (DAUNOMYCIN HCL) | 0.0090 | 0.0026 | 0.0069 |
| TOPIRAMATE | 0.1433 | 0.1725 | 0.0070 |
| ZILEUTON | 0.1401 | 0.1683 | 0.0071 |
| SULFASALAZINE (AZULFIDINE) | 0.1489 | 0.1793 | 0.0072 |
| KETOPROFEN (ACTRON) | 0.1357 | 0.1625 | 0.0074 |
| ZOLMITRIPTAN (ZOMIG) | 0.1521 | 0.1832 | 0.0074 |
| LOMUSTINE (CEENU) | 0.1498 | 0.1802 | 0.0074 |
| TAZAROTENE (AVAGE) | 0.1426 | 0.1710 | 0.0075 |
| BESIFLOXACIN HCL (BESIVANCE) | 0.0096 | 0.0025 | 0.0076 |
| LENALIDOMIDE | 0.1447 | 0.1734 | 0.0077 |
| ROXATIDINE ACETATE HCL | 0.1424 | 0.1702 | 0.0079 |
| CARBAZOCHROME SODIUM SULFONATE | 0.1591 | 0.1912 | 0.0080 |
| RIFAPENTINE (PRIFTIN) | 0.0115 | 0.0044 | 0.0080 |
| OTILONIUM BROMIDE | 0.0106 | 0.0031 | 0.0081 |
| SOTALOL (BETAPACE) | 0.1558 | 0.1868 | 0.0082 |
| CATHARANTHINE | 0.1566 | 0.1879 | 0.0082 |
| TRIFLURIDINE (VIROPTIC) | 0.1446 | 0.1726 | 0.0082 |
| SOLIFENACIN SUCCINATE | 0.1514 | 0.1812 | 0.0082 |
| TRIMETAZIDINE DIHCL | 0.1527 | 0.1827 | 0.0084 |
| SULFAMETER (BAYRENA) | 0.1581 | 0.1895 | 0.0084 |
| EPALRESTAT | 0.1633 | 0.1960 | 0.0085 |
| FLUMAZENIL | 0.1486 | 0.1774 | 0.0085 |
| LOVASTATIN (MEVACOR) | 0.1431 | 0.1703 | 0.0085 |
| MEPTAZINOL HCL | 0.1550 | 0.1853 | 0.0085 |
| CHLOROTHIAZIDE | 0.1485 | 0.1771 | 0.0086 |
| ASPIRIN (ACETYLSALICYLIC ACID) | 0.1581 | 0.1893 | 0.0086 |
| IMATINIB (GLEEVEC) | 0.1476 | 0.1759 | 0.0086 |
| DIPHENHYDRAMINE HCL (BENADRYL) | 0.1424 | 0.1693 | 0.0087 |
| ISONIAZID (TUBIZID) | 0.1548 | 0.1849 | 0.0087 |
| ECONAZOLE NITRATE (SPECTAZOLE) | 0.0097 | 0.0012 | 0.0087 |
| VIDARABINE (VIRA-A) | 0.1480 | 0.1762 | 0.0088 |
| GEMFIBROZIL (LOPID) | 0.1338 | 0.1582 | 0.0088 |
| REPAGLINIDE | 0.1392 | 0.1650 | 0.0089 |
| 2-THIOURACIL | 0.1526 | 0.1817 | 0.0090 |
| IRSOGLADINE | 0.1375 | 0.1627 | 0.0090 |
| GLIMEPIRIDE | 0.1552 | 0.1850 | 0.0090 |
| TRANEXAMIC ACID (TRANSAMIN) | 0.1448 | 0.1718 | 0.0091 |
| RESVERATROL | 0.1438 | 0.1703 | 0.0093 |
| PREDNISOLONE ACETATE (OMNIPRED) | 0.1614 | 0.1924 | 0.0094 |
| RISEDRONIC ACID (ACTONEL) | 0.1511 | 0.1792 | 0.0095 |
| TRIFLUSAL | 0.1647 | 0.1965 | 0.0095 |
| LAMIVUDINE (EPIVIR) | 0.1610 | 0.1917 | 0.0095 |
| FLUCYTOSINE (ANCOBON) | 0.1352 | 0.1590 | 0.0095 |
| RIMONABANT (SR141716) | 0.1584 | 0.1884 | 0.0096 |
| L-GLUTAMINE | 0.1543 | 0.1831 | 0.0096 |
| ALBENDAZOLE (ALBENZA) | 0.1398 | 0.1647 | 0.0097 |
| VILDAGLIPTIN (LAF-237) | 0.1569 | 0.1862 | 0.0098 |
| SULFAMERAZINE | 0.1444 | 0.1702 | 0.0099 |
| GESTODENE | 0.1559 | 0.1848 | 0.0099 |
| ROSIGLITAZONE MALEATE | 0.1495 | 0.1764 | 0.0101 |
| AZATADINE DIMALEATE | 0.1605 | 0.1904 | 0.0101 |
| HYDROXYUREA (CYTODROX) | 0.1415 | 0.1661 | 0.0103 |
| RANOLAZINE (RANEXA) | 0.1460 | 0.1718 | 0.0103 |
| UNKNOWN | 0.1505 | 0.1772 | 0.0105 |
| GINKGOLIDE A | 0.1681 | 0.1995 | 0.0105 |
| UNKNOWN | 0.1450 | 0.1703 | 0.0105 |
| GADODIAMIDE (OMNISCAN) | 0.1493 | 0.1755 | 0.0106 |
| IMIQUIMOD | 0.1523 | 0.1793 | 0.0106 |
| AMIKACIN SULFATE | 0.1539 | 0.1812 | 0.0108 |
| ALVERINE CITRATE | 0.1542 | 0.1816 | 0.0108 |
| CLEMASTINE FUMARATE | 0.1406 | 0.1642 | 0.0109 |
| FENOPROFEN CALCIUM | 0.1486 | 0.1742 | 0.0109 |
| OSELTAMIVIR PHOSPHATE (TAMIFLU) | 0.1620 | 0.1912 | 0.0110 |
| BEPOTASTINE BESILATE | 0.1534 | 0.1800 | 0.0112 |
| PIROXICAM (FELDENE) | 0.1384 | 0.1608 | 0.0114 |
| DEFLAZACORT (CALCORT) | 0.1502 | 0.1757 | 0.0114 |
| MONOBENZONE (BENOQUIN) | 0.1425 | 0.1658 | 0.0115 |
| TRIAMCINOLONE ACETONIDE | 0.1359 | 0.1572 | 0.0117 |
| GANCICLOVIR | 0.1460 | 0.1699 | 0.0118 |
| VINPOCETINE (CAVINTON) | 0.1586 | 0.1859 | 0.0118 |
| UNKNOWN | 0.1497 | 0.1745 | 0.0118 |
| VALSARTAN (DIOVAN) | 0.1446 | 0.1680 | 0.0119 |
| PAROXETINE HCL | 0.1563 | 0.1827 | 0.0119 |
| NEFIRACETAM (TRANSLON) | 0.1677 | 0.1970 | 0.0120 |
| KETOROLAC (TORADOL) | 0.1376 | 0.1584 | 0.0124 |
| PIMOBENDAN (VETMEDIN) | 0.1572 | 0.1833 | 0.0124 |
| IFOSFAMIDE | 0.1701 | 0.1995 | 0.0124 |
| TALC | 0.1427 | 0.1648 | 0.0125 |
| PANCURONIUM (PAVULON) | 0.1713 | 0.2009 | 0.0126 |
| CRYSTAL VIOLET | 0.1437 | 0.1660 | 0.0126 |
| SULFADIAZINE | 0.1541 | 0.1791 | 0.0126 |
| BENSERAZIDE | 0.1541 | 0.1791 | 0.0126 |
| PMSF (PHENYLMETHYLSULFONYL FLUORIDE) | 0.1655 | 0.1934 | 0.0127 |
| TIAMULIN HYD. PHSOP. | 0.1141 | 0.1283 | 0.0127 |
| SUPLATAST TOSYLATE | 0.1562 | 0.1814 | 0.0128 |
| TROPISETRON | 0.1432 | 0.1647 | 0.0130 |
| FLUVOXAMINE MALEATE | 0.1490 | 0.1719 | 0.0131 |
| NALOXONE HCL | 0.1547 | 0.1791 | 0.0132 |
| MESNA (UROMITEXAN, MESNEX) | 0.1588 | 0.1842 | 0.0133 |
| OLOPATADINE HYDROCHLORIDE (OPATANOL) | 0.1504 | 0.1736 | 0.0133 |
| BINDARIT | 0.1591 | 0.1846 | 0.0133 |
| MYCOPHENOLATE MOFETIL (CELLCEPT) | 0.1303 | 0.1481 | 0.0133 |
| ACIPIMOX | 0.1424 | 0.1634 | 0.0133 |
| HALOPERIDOL (HALDOL) | 0.1411 | 0.1617 | 0.0134 |
| INULIN | 0.1629 | 0.1893 | 0.0134 |
| ALFACALCIDOL | 0.1361 | 0.1552 | 0.0135 |
| PITAVASTATIN CALCIUM (LIVALO) | 0.1486 | 0.1710 | 0.0135 |
| VICRIVIROC MALATE | 0.1644 | 0.1907 | 0.0137 |
| CANDESARTAN (ATACAND) | 0.1482 | 0.1702 | 0.0138 |
| DAIDZEIN | 0.1424 | 0.1627 | 0.0138 |
| PALONOSETRON HCL | 0.1570 | 0.1811 | 0.0139 |
| ROSUVASTATIN CALCIUM (CRESTOR) | 0.1778 | 0.2074 | 0.0139 |
| PROGESTERONE (PROMETRIUM) | 0.1623 | 0.1878 | 0.0139 |
| GENIPOSIDIC ACID | 0.1633 | 0.1889 | 0.0140 |
| CHLOROXINE | 0.1505 | 0.1727 | 0.0140 |
| NICORANDIL (IKOREL) | 0.1582 | 0.1824 | 0.0141 |
| CABAZITAXEL (JEVTANA) | 0.1459 | 0.1668 | 0.0142 |
| DONEPEZIL HCL (ARICEPT) | 0.1594 | 0.1837 | 0.0143 |
| DILTIAZEM HCL (TIAZAC) | 0.1627 | 0.1878 | 0.0144 |
| PRAMIRACETAM | 0.1674 | 0.1935 | 0.0145 |
| SULFANILAMIDE | 0.1527 | 0.1747 | 0.0146 |
| FEBUXOSTAT (ULORIC) | 0.1475 | 0.1677 | 0.0151 |
| CIPROFLOXACIN (CIPRO) | 0.1399 | 0.1579 | 0.0152 |
| ALLOPURINOL SODIUM (ALOPRIM) | 0.1562 | 0.1784 | 0.0153 |
| METHOCARBAMOL (ROBAXIN) | 0.1587 | 0.1814 | 0.0154 |
| ACADESINE | 0.1435 | 0.1621 | 0.0154 |
| CARBOPLATIN | 0.1710 | 0.1969 | 0.0155 |
| FLEROXACIN (QUINODIS) | 0.1520 | 0.1726 | 0.0156 |
| SULBACTAM SODIUM (UNASYN) | 0.1609 | 0.1840 | 0.0156 |
| TENOXICAM (MOBIFLEX) | 0.1546 | 0.1759 | 0.0157 |
| TROPICAMIDE | 0.1695 | 0.1947 | 0.0157 |
| PITOFENONE HCL | 0.1535 | 0.1742 | 0.0158 |
| RACECADOTRIL (ACETORPHAN) | 0.1715 | 0.1969 | 0.0159 |
| PYRAZINAMIDE (PYRAZINOIC ACID AMIDE) | 0.1470 | 0.1659 | 0.0159 |
| SPARFLOXACIN | 0.0875 | 0.0906 | 0.0159 |
| AZELNIDIPINE | 0.1557 | 0.1770 | 0.0160 |
| VENLAFAXINE | 0.1512 | 0.1712 | 0.0160 |
| EPLERENONE | 0.1591 | 0.1807 | 0.0163 |
| URAPIDIL HCL | 0.1739 | 0.1993 | 0.0165 |
| LACIDIPINE (LACIPIL, MOTENS) | 0.1577 | 0.1788 | 0.0165 |
| BUDESONIDE | 0.1608 | 0.1826 | 0.0165 |
| CARBENICILLIN DISODIUM | 0.1468 | 0.1648 | 0.0166 |
| FLUCONAZOLE | 0.1504 | 0.1692 | 0.0167 |
| CARBIDOPA | 0.1466 | 0.1643 | 0.0168 |
| SULFISOXAZOLE | 0.1439 | 0.1609 | 0.0168 |
| METHIMAZOLE (TAPAZOLE, NORTHYX) | 0.1431 | 0.1599 | 0.0168 |
| ZONISAMIDE | 0.1535 | 0.1731 | 0.0168 |
| MALOTILATE | 0.1569 | 0.1773 | 0.0168 |
| PROBUCOL | 0.1651 | 0.1876 | 0.0169 |
| POTASSIUM IODIDE | 0.1421 | 0.1584 | 0.0169 |
| NIZATIDINE | 0.1498 | 0.1682 | 0.0170 |
| VARENICLINE TARTRATE | 0.1577 | 0.1781 | 0.0170 |
| THIOGUANINE | 0.1563 | 0.1763 | 0.0170 |
| ELLAGIC ACID | 0.1670 | 0.1898 | 0.0171 |
| ORNIDAZOLE | 0.1539 | 0.1731 | 0.0171 |
| CIMETIDINE (TAGAMET) | 0.1466 | 0.1635 | 0.0174 |
| CARBAMAZEPINE (CARBATROL) | 0.1558 | 0.1750 | 0.0175 |
| RANITIDINE (ZANTAC) | 0.1478 | 0.1646 | 0.0178 |
| AXITINIB | 0.1585 | 0.1775 | 0.0182 |
| ULIPRISTAL | 0.1590 | 0.1781 | 0.0182 |
| ETHIONAMIDE | 0.1452 | 0.1606 | 0.0183 |
| IBANDRONATE SODIUM | 0.1623 | 0.1820 | 0.0185 |
| LURASIDONE HCL | 0.1507 | 0.1672 | 0.0186 |
| ROCURONIUM BROMIDE | 0.1695 | 0.1909 | 0.0187 |
| NEOSTIGMINE BROMIDE (PROSTIGMIN) | 0.1575 | 0.1755 | 0.0189 |
| PAMIDRONATE DISODIUM | 0.1456 | 0.1603 | 0.0189 |
| 5-AMINOLEVULINIC ACID HYDROCHLORIDE | 0.1517 | 0.1680 | 0.0190 |
| BETA CAROTENE | 0.1659 | 0.1859 | 0.0190 |
| UBENIMEX (BESTATIN) | 0.1650 | 0.1845 | 0.0192 |
| CISATRACURIUM BESYLATE (NIMBEX) | 0.1669 | 0.1869 | 0.0192 |
| AMINOGLUTETHIMIDE (CYTADREN) | 0.1727 | 0.1942 | 0.0193 |
| MOMETASONE FUROATE | 0.1607 | 0.1791 | 0.0193 |
| ACYCLOVIR (ACICLOVIR) | 0.1430 | 0.1566 | 0.0193 |
| LETROZOLE | 0.1547 | 0.1714 | 0.0193 |
| LORNOXICAM (XEFO) | 0.1449 | 0.1589 | 0.0194 |
| NYSTATIN (MYCOSTATIN) | 0.1651 | 0.1844 | 0.0194 |
| CLINDAMYCIN PHOSPHATE | 0.1560 | 0.1729 | 0.0194 |
| CYTARABINE | 0.1577 | 0.1750 | 0.0195 |
| SUMATRIPTAN SUCCINATE | 0.1657 | 0.1848 | 0.0197 |
| PERAMIVIR TRIHYDRATE | 0.1680 | 0.1877 | 0.0197 |
| GIMERACIL | 0.1790 | 0.2016 | 0.0197 |
| MOCLOBEMIDE | 0.1618 | 0.1797 | 0.0198 |
| AZILSARTAN MEDOXOMIL (TAK-491) | 0.1499 | 0.1644 | 0.0199 |
| RASAGILINE MESYLATE | 0.1726 | 0.1932 | 0.0200 |
| GLIPIZIDE (GLUCOTROL) | 0.1647 | 0.1830 | 0.0201 |
| DROSPIRENONE | 0.1646 | 0.1829 | 0.0201 |
| GRANISETRON HCL | 0.1707 | 0.1903 | 0.0203 |
| CLAFEN (CYCLOPHOSPHAMIDE) | 0.1662 | 0.1846 | 0.0204 |
| BIMATOPROST | 0.1590 | 0.1754 | 0.0205 |
| NORADRENALINE BITARTRATE MONOHYDRATE (LEVOPHED) | 0.1667 | 0.1850 | 0.0206 |
| ESTRADIOL | 0.1595 | 0.1757 | 0.0206 |
| FORMOTEROL HEMIFUMARATE | 0.1506 | 0.1645 | 0.0206 |
| DEXTROSE (D-GLUCOSE) | 0.1639 | 0.1810 | 0.0209 |
| BLONANSERIN (LONASEN) | 0.1689 | 0.1872 | 0.0210 |
| MESTRANOL | 0.1659 | 0.1834 | 0.0211 |
| RISEDRONATE SODIUM | 0.1677 | 0.1855 | 0.0211 |
| BUFLOMEDIL HCL | 0.1698 | 0.1881 | 0.0212 |
| BENDAMUSTINE HCL | 0.1677 | 0.1852 | 0.0214 |
| HYDROCHLOROTHIAZIDE | 0.1742 | 0.1934 | 0.0214 |
| INDOMETHACIN (INDOCID, INDOCIN) | 0.1568 | 0.1714 | 0.0214 |
| ZIPRASIDONE HYDROCHLORIDE | 0.1558 | 0.1700 | 0.0215 |
| IRINOTECAN | 0.1600 | 0.1751 | 0.0216 |
| LINAGLIPTIN (BI-1356) | 0.1655 | 0.1821 | 0.0217 |
| OXCARBAZEPINE | 0.1618 | 0.1773 | 0.0217 |
| TORASEMIDE | 0.1606 | 0.1758 | 0.0218 |
| 2-METHOXYESTRADIOL | 0.1651 | 0.1815 | 0.0218 |
| MEDETOMIDINE HCL | 0.1628 | 0.1784 | 0.0219 |
| DACARBAZINE (DTIC-DOME) | 0.1502 | 0.1623 | 0.0220 |
| RACTOPAMINE HCL | 0.1638 | 0.1794 | 0.0221 |
| TRIMETHOPRIM | 0.0266 | 0.0057 | 0.0221 |
| VORINOSTAT (SAHA) | 0.1756 | 0.1941 | 0.0223 |
| TIANEPTINE SODIUM | 0.1904 | 0.2128 | 0.0223 |
| IDOXURIDINE | 0.1547 | 0.1676 | 0.0223 |
| BUFEXAMAC | 0.1655 | 0.1811 | 0.0224 |
| AMPRENAVIR (AGENERASE) | 0.1742 | 0.1916 | 0.0229 |
| LANSOPRAZOLE | 0.1588 | 0.1719 | 0.0230 |
| MEMANTINE HCL (NAMENDA) | 0.1828 | 0.2023 | 0.0230 |
| DROXIDOPA (L-DOPS) | 0.1635 | 0.1775 | 0.0233 |
| TOLBUTAMIDE | 0.1529 | 0.1638 | 0.0235 |
| GUAIFENESIN (GUAIPHENESIN) | 0.1476 | 0.1568 | 0.0237 |
| DICHLORPHENAMIDE (DICLOFENAMIDE) | 0.1849 | 0.2040 | 0.0238 |
| MYCOPHENOLATE MOFETIL | 0.1534 | 0.1640 | 0.0239 |
| ATROPINE | 0.1691 | 0.1838 | 0.0239 |
| MOGUISTEINE | 0.1803 | 0.1978 | 0.0240 |
| ASPARTAME | 0.1666 | 0.1804 | 0.0241 |
| MOSAPRIDE CITRATE | 0.1704 | 0.1851 | 0.0242 |
| ABITREXATE (METHOTREXATE) | 0.1528 | 0.1628 | 0.0242 |
| BUPIVACAINE HYDROCHLORIDE (MARCAIN) | 0.1621 | 0.1745 | 0.0243 |
| DAPTOMYCIN | 0.1599 | 0.1717 | 0.0243 |
| CEFDITOREN PIVOXIL | 0.1612 | 0.1732 | 0.0243 |
| PRANOPROFEN | 0.1577 | 0.1688 | 0.0244 |
| AMILORIDE HYDROCHLORIDE (MIDAMOR) | 0.1660 | 0.1792 | 0.0245 |
| RAMIPRIL | 0.1913 | 0.2108 | 0.0247 |
| OZAGREL HCL | 0.1641 | 0.1763 | 0.0248 |
| OXALIPLATIN (ELOXATIN) | 0.1603 | 0.1715 | 0.0248 |
| FENBENDAZOLE (PANACUR) | 0.1646 | 0.1765 | 0.0252 |
| DOCETAXEL (TAXOTERE) | 0.1732 | 0.1869 | 0.0255 |
| LAMOTRIGINE | 0.1750 | 0.1890 | 0.0256 |
| PHENTOLAMINE MESILATE | 0.1666 | 0.1783 | 0.0257 |
| CHLORMEZANONE (TRANCOPAL) | 0.1597 | 0.1690 | 0.0262 |
| GENIPOSIDE | 0.1805 | 0.1952 | 0.0263 |
| NILVADIPINE (ARC029) | 0.1732 | 0.1855 | 0.0266 |
| CARVEDILOL | 0.1634 | 0.1731 | 0.0266 |
| BETHANECHOL CHLORIDE | 0.1734 | 0.1853 | 0.0269 |
| ISOTRETINOIN | 0.1646 | 0.1741 | 0.0270 |
| CYTIDINE | 0.1669 | 0.1768 | 0.0272 |
| VALACICLOVIR HCL | 0.1687 | 0.1788 | 0.0274 |
| TIGECYCLINE | 0.1648 | 0.1737 | 0.0276 |
| MELATONIN | 0.1742 | 0.1856 | 0.0276 |
| BISOPROLOL | 0.1684 | 0.1782 | 0.0277 |
| ONDANSETRON HCL (ZOFRAN) | 0.1723 | 0.1829 | 0.0278 |
| ZOLEDRONIC ACID (ZOLEDRONATE) | 0.1723 | 0.1823 | 0.0283 |
| ALTRETAMINE (HEXALEN) | 0.1826 | 0.1953 | 0.0283 |
| IRBESARTAN (AVAPRO) | 0.1647 | 0.1726 | 0.0284 |
| ARTICAINE HCL | 0.1475 | 0.1508 | 0.0284 |
| PAZOPANIB HCL | 0.1718 | 0.1813 | 0.0285 |
| VINBLASTINE | 0.1875 | 0.2010 | 0.0286 |
| NISOLDIPINE (SULAR) | 0.1801 | 0.1916 | 0.0287 |
| MEGLUMINE | 0.1709 | 0.1799 | 0.0287 |
| MERCAPTOPURINE | 0.1996 | 0.2163 | 0.0288 |
| VARDENAFIL (VIVANZA) | 0.1731 | 0.1826 | 0.0288 |
| PEMETREXED | 0.1686 | 0.1758 | 0.0297 |
| EMTRICITABINE (EMTRIVA) | 0.1951 | 0.2090 | 0.0300 |
| MOROXYDINE | 0.1723 | 0.1802 | 0.0300 |
| ROFECOXIB (VIOXX) | 0.1717 | 0.1794 | 0.0300 |
| ABIRATERONE (CB-7598) | 0.1707 | 0.1780 | 0.0300 |
| ESTRADIOL VALERATE | 0.1681 | 0.1747 | 0.0301 |
| MILRINONE (PRIMACOR) | 0.1755 | 0.1835 | 0.0305 |
| BUMETANIDE | 0.1730 | 0.1802 | 0.0306 |
| LEVOSIMENDAN | 0.1665 | 0.1719 | 0.0307 |
| BUSULFAN (MYLERAN, BUSULFEX) | 0.1742 | 0.1807 | 0.0315 |
| AZILSARTAN (TAK-536) | 0.1666 | 0.1709 | 0.0316 |
| CLADRIBINE | 0.1763 | 0.1831 | 0.0316 |
| FLURBIPROFEN (ANSAID) | 0.1530 | 0.1534 | 0.0319 |
| THALIDOMIDE | 0.1731 | 0.1781 | 0.0324 |
| NEPAFENAC | 0.1779 | 0.1838 | 0.0327 |
| LEVAMISOLE HYDROCHLORIDE (ERGAMISOL) | 0.1958 | 0.2051 | 0.0338 |
| SODIUM SALICYLATE | 0.1785 | 0.1831 | 0.0338 |
| CINEPAZIDE MALEATE | 0.1739 | 0.1766 | 0.0344 |
| BETAHISTINE 2HCL | 0.1831 | 0.1882 | 0.0344 |
| BETAMETHASONE DIPROPIONATE (DIPROLENE) | 0.1817 | 0.1862 | 0.0346 |
| AZLOCILLIN SODIUM SALT | 0.1698 | 0.1704 | 0.0352 |
| DEXAMETHASONE | 0.1742 | 0.1756 | 0.0355 |
| METRONIDAZOLE (FLAGYL) | 0.1777 | 0.1800 | 0.0355 |
| SODIUM ORTHOVANADATE | 0.1810 | 0.1842 | 0.0355 |
| METHYLPREDNISOLONE | 0.1750 | 0.1758 | 0.0362 |
| VANDETANIB (ZACTIMA) | 0.1880 | 0.1922 | 0.0362 |
| RUFINAMIDE (BANZEL) | 0.1933 | 0.1986 | 0.0364 |
| TOPOTECAN HCL | 0.1762 | 0.1768 | 0.0365 |
| AMIDOPYRINE | 0.1804 | 0.1819 | 0.0367 |
| RIMANTADINE (FLUMADINE) | 0.1689 | 0.1673 | 0.0367 |
| SODIUM BUTYRATE | 0.1834 | 0.1856 | 0.0368 |
| FLUTICASONE PROPIONATE (FLONASE, VERAMYST) | 0.1701 | 0.1687 | 0.0368 |
| MYCOPHENOLIC (MYCOPHENOLATE) | 0.1339 | 0.1228 | 0.0369 |
| AMPIROXICAM | 0.1998 | 0.2062 | 0.0369 |
| RAMELTEON (TAK-375) | 0.1764 | 0.1765 | 0.0370 |
| KETOCONAZOLE | 0.1770 | 0.1760 | 0.0380 |
| ANIRACETAM | 0.1758 | 0.1743 | 0.0381 |
| SULPHADIMETHOXINE | 0.1665 | 0.1613 | 0.0391 |
| PREDNISOLONE (HYDRORETROCORTINE) | 0.1797 | 0.1778 | 0.0392 |
| LEVOFLOXACIN (LEVAQUIN) | 0.0429 | 0.0034 | 0.0402 |
| TAURINE | 0.1770 | 0.1727 | 0.0406 |
| MICONAZOLE NITRATE | 0.0442 | 0.0041 | 0.0410 |
| ORPHENADRINE CITRATE (NORFLEX) | 0.1906 | 0.1887 | 0.0416 |
| MESALAMINE (LIALDA) | 0.1692 | 0.1615 | 0.0416 |
| SUNITINIB MALATE (SUTENT) | 0.1825 | 0.1779 | 0.0420 |
| BENAZEPRIL HYDROCHLORIDE | 0.1950 | 0.1933 | 0.0423 |
| CLINDAMYCIN PALMITATE HCL | 0.1239 | 0.1032 | 0.0424 |
| GENIPIN | 0.1943 | 0.1921 | 0.0425 |
| ASENAPINE | 0.2129 | 0.2156 | 0.0426 |
| STREPTOZOTOCIN (ZANOSAR) | 0.1714 | 0.1629 | 0.0427 |
| METHAZOLASTONE | 0.1683 | 0.1585 | 0.0431 |
| CLOFARABINE | 0.1788 | 0.1715 | 0.0432 |
| AMIKACIN HYDRATE | 0.1850 | 0.1794 | 0.0433 |
| FLUDARABINE PHOSPHATE (FLUDARA) | 0.1849 | 0.1786 | 0.0438 |
| DISULFIRAM (ANTABUSE) | 0.1163 | 0.0917 | 0.0438 |
| DECITABINE | 0.1586 | 0.1444 | 0.0445 |
| NIMESULIDE | 0.1557 | 0.1403 | 0.0449 |
| AGOMELATINE | 0.1765 | 0.1665 | 0.0450 |
| HYDROCORTISONE (CORTISOL) | 0.1738 | 0.1629 | 0.0451 |
| PRASUGREL (EFFIENT) | 0.1875 | 0.1799 | 0.0453 |
| IPRATROPIUM BROMIDE | 0.1796 | 0.1698 | 0.0455 |
| VITAMIN B12 | 0.1964 | 0.1907 | 0.0457 |
| FLUMEQUINE | 0.0811 | 0.0446 | 0.0459 |
| ATOVAQUONE (ATAVAQUONE) | 0.1928 | 0.1843 | 0.0472 |
| ETHISTERONE | 0.1929 | 0.1839 | 0.0476 |
| AMINOPHYLLINE (TRUPHYLLINE) | 0.1950 | 0.1864 | 0.0477 |
| MARAVIROC | 0.1621 | 0.1442 | 0.0481 |
| AMANTADINE HYDROCHLORIDE (SYMMETREL) | 0.1936 | 0.1838 | 0.0484 |
| ACARBOSE | 0.1880 | 0.1762 | 0.0488 |
| DIPYRIDAMOLE (PERSANTINE) | 0.1901 | 0.1759 | 0.0511 |
| DESONIDE | 0.1822 | 0.1650 | 0.0519 |
| SARAFLOXACIN HCL | 0.0767 | 0.0312 | 0.0521 |
| ZALCITABINE | 0.2033 | 0.1913 | 0.0522 |
| SULFATHIAZOLE | 0.1943 | 0.1789 | 0.0529 |
| EDARAVONE (MCI-186) | 0.1803 | 0.1596 | 0.0543 |
| LORATADINE | 0.2032 | 0.1874 | 0.0552 |
| AMPICILLIN SODIUM | 0.1461 | 0.1115 | 0.0580 |
| CHLORAMPHENICOL (CHLOROMYCETIN) | 0.2079 | 0.1875 | 0.0598 |
| VECURONIUM BROMIDE | 0.2210 | 0.2038 | 0.0600 |
| AMOXICILLIN (AMOXYCILLIN) | 0.1601 | 0.1265 | 0.0601 |
| AMINOCAPROIC ACID (AMICAR) | 0.1965 | 0.1713 | 0.0612 |
| VINCRISTINE | 0.1960 | 0.1698 | 0.0619 |
| PROCARBAZINE HCL (MATULANE) | 0.1712 | 0.1373 | 0.0627 |
| SORAFENIB (NEXAVAR) | 0.0885 | 0.0317 | 0.0634 |
| METHOXSALEN (OXSORALEN) | 0.1899 | 0.1599 | 0.0635 |
| BEXAROTENE | 0.0689 | 0.0026 | 0.0668 |
| NORFLOXACIN (NORXACIN) | 0.1341 | 0.0818 | 0.0695 |
| PROPYLTHIOURACIL | 0.1753 | 0.1265 | 0.0754 |
| BALOFLOXACIN | 0.1423 | 0.0763 | 0.0820 |
| AZTREONAM (AZACTAM, CAYSTON) | 0.1383 | 0.0675 | 0.0850 |
| AZTREONAM | 0.1643 | 0.0975 | 0.0873 |
| RAFOXINIDE | 0.0969 | 0.0065 | 0.0917 |
| VANCOMYCIN HCL (VANCOCIN) | 0.0957 | 0.0043 | 0.0923 |
| FROPENEM | 0.1093 | 0.0046 | 0.1056 |
| CEFUROXIME AXETIL | 0.1594 | 0.0589 | 0.1129 |
| TENIPOSIDE | 0.1524 | 0.0500 | 0.1130 |
| CARMOFUR | 0.1520 | 0.0473 | 0.1147 |
| BIFONAZOLE | 0.1503 | 0.0375 | 0.1207 |
| RUFLOXACIN HCL | 0.1564 | 0.0414 | 0.1237 |
| CEFATRIZINE | 0.1530 | 0.0331 | 0.1268 |
| DORIPENEM HYDRATE | 0.1558 | 0.0204 | 0.1397 |
| CEFOSELIS SULFATE | 0.1443 | 0.0041 | 0.1411 |
| TELITHROMYCIN | 0.1513 | 0.0125 | 0.1414 |
| CEFDINIR (OMNICEF) | 0.1456 | 0.0049 | 0.1417 |
| OXACILLIN SODIUM MONOHYDRATE | 0.1446 | 0.0024 | 0.1427 |
| CEFOPERAZONE (CEFOBID) | 0.1479 | 0.0021 | 0.1462 |
| TOTAROL | 0.1548 | 0.0050 | 0.1509 |
| MEROPENEM | 0.1731 | 0.0263 | 0.1523 |
| TOSUFLOXACIN TOSYLATE | 0.1553 | 0.0031 | 0.1529 |
| AZITHROMYCIN | 0.1639 | 0.0028 | 0.1617 |
| CEFACLOR (CECLOR) | 0.1743 | 0.0014 | 0.1732 |
| TEBIPENEM PIVOXIL (L-084) | 0.1897 | 0.0025 | 0.1877 |
| RIFAXIMIN | 0.2113 | 0.0042 | 0.2080 |
| ROXITHROMYCIN | 0.2247 | 0.0142 | 0.2135 |
