# Supplementary material for: Repurposing drugs with specific activity against L-form bacteria
Source: Front Microbiol. 2023 Apr 4;14:1097413. doi: 10.3389/fmicb.2023.1097413 (PMC10110866; doi:10.3389/fmicb.2023.1097413)
Supplement: Supplementary file 2 [file Table_2.docx]

**Supplementary Table S2. Second screen of the FDA compound set.**

| **Compound** | **AUC L-forms** | **AUC Walled cells** | **Residuals** |
| --- | --- | --- | --- |
| MANIDIPINE (MANYPER) | 0.0072 | 0.1873 | -0.1440 |
| MECLIZINE 2HCL | 0.0083 | 0.1637 | -0.1238 |
| ACETYLCHOLINE CHLORIDE | 0.1359 | 0.3205 | -0.1228 |
| FLUNARIZINE 2HCL | 0.0054 | 0.1584 | -0.1224 |
| TRIFLUOPERAZINE 2HCL | 0.0181 | 0.1659 | -0.1158 |
| ETRAVIRINE (TMC125) | 0.0105 | 0.1499 | -0.1105 |
| FOSAPREPITANT DIMEGLUMINE | 0.0093 | 0.1443 | -0.1071 |
| DRONEDARONE HCL (MULTAQ) | 0.0064 | 0.1282 | -0.0971 |
| BUTENAFINE HCL | 0.0453 | 0.1733 | -0.0946 |
| CLOMIPRAMINE HYDROCHLORIDE (ANAFRANIL) | 0.1265 | 0.2705 | -0.0918 |
| NICLOSAMIDE (NICLOCIDE) | 0.0103 | 0.1213 | -0.0876 |
| AMIODARONE HCL | 0.0096 | 0.1065 | -0.0763 |
| DICLOFENAC POTASSIUM | 0.0623 | 0.1543 | -0.0622 |
| DICLOFENAC | 0.0595 | 0.1480 | -0.0600 |
| NIFLUMIC ACID | 0.0384 | 0.1203 | -0.0587 |
| TOLNAFTATE | 0.1293 | 0.2321 | -0.0580 |
| TIOCONAZOLE | 0.0062 | 0.0774 | -0.0563 |
| TOBRAMYCIN | 0.0078 | 0.0777 | -0.0549 |
| ZAFIRLUKAST (ACCOLATE) | 0.0074 | 0.0740 | -0.0524 |
| METHACYCLINE HYDROCHLORIDE (PHYSIOMYCINE) | 0.0619 | 0.1409 | -0.0518 |
| TERBINAFINE (LAMISIL, TERBINEX) | 0.0768 | 0.1582 | -0.0509 |
| LIRANAFTATE | 0.0869 | 0.1665 | -0.0475 |
| DOXIFLURIDINE | 0.0760 | 0.1496 | -0.0448 |
| PRAMIPEXOLE DIHYDROCHLORIDE MONOHYDRATE | 0.1435 | 0.2327 | -0.0443 |
| METHENAMINE (MANDELAMINE) | 0.1484 | 0.2363 | -0.0423 |
| DICLAZURIL | 0.0140 | 0.0695 | -0.0421 |
| UNKNOWN | 0.0060 | 0.0587 | -0.0413 |
| AZELASTINE HYDROCHLORIDE (ASTELIN) | 0.1260 | 0.2050 | -0.0394 |
| GABAPENTIN (NEURONTIN) | 0.1271 | 0.2057 | -0.0389 |
| SARAFLOXACIN HCL | 0.0060 | 0.0520 | -0.0360 |
| ISOPRENALINE HYDROCHLORIDE | 0.1269 | 0.1988 | -0.0335 |
| HYGROMYCIN B | 0.1023 | 0.1672 | -0.0327 |
| L-THYROXINE | 0.1014 | 0.1658 | -0.0324 |
| MITOTANE (LYSODREN) | 0.1346 | 0.2047 | -0.0306 |
| ALBENDAZOLE OXIDE (RICOBENDAZOLE) | 0.1644 | 0.2403 | -0.0296 |
| VALPROIC ACID SODIUM SALT (SODIUM VALPROATE) | 0.1534 | 0.2260 | -0.0290 |
| THIAMPHENICOL (THIOPHENICOL) | 0.0215 | 0.0613 | -0.0280 |
| UNKNOWN | 0.0980 | 0.1560 | -0.0279 |
| ATORVASTATIN CALCIUM (LIPITOR) | 0.1099 | 0.1700 | -0.0274 |
| FENTICONAZOLE NITRATE | 0.0056 | 0.0408 | -0.0273 |
| MAPROTILINE HYDROCHLORIDE | 0.1410 | 0.2078 | -0.0267 |
| AZACITIDINE (VIDAZA) | 0.1451 | 0.2122 | -0.0262 |
| SPECTINOMYCIN HYDROCHLORIDE | 0.1046 | 0.1601 | -0.0246 |
| TOLTERODINE TARTRATE (DETROL LA) | 0.1287 | 0.1889 | -0.0238 |
| PIMECROLIMUS | 0.1085 | 0.1635 | -0.0234 |
| DULOXETINE HCL (CYMBALTA) | 0.1176 | 0.1736 | -0.0225 |
| RESERPINE | 0.1072 | 0.1595 | -0.0216 |
| NITRENDIPINE | 0.1252 | 0.1818 | -0.0215 |
| FK-506 (TACROLIMUS) | 0.1019 | 0.1526 | -0.0213 |
| NIFEDIPINE (ADALAT) | 0.1280 | 0.1849 | -0.0212 |
| NADIFLOXACIN | 0.0046 | 0.0313 | -0.0206 |
| PRANLUKAST | 0.1143 | 0.1670 | -0.0205 |
| DOPAMINE HYDROCHLORIDE (INOTROPIN) | 0.1295 | 0.1842 | -0.0191 |
| MECARBINATE | 0.1218 | 0.1743 | -0.0189 |
| DIETHYLSTILBESTROL (STILBESTROL) | 0.0943 | 0.1399 | -0.0186 |
| PROPAFENONE (RYTMONORM) | 0.1308 | 0.1844 | -0.0181 |
| INDACATEROL MALEATE | 0.1144 | 0.1640 | -0.0179 |
| ITRACONAZOLE (SPORANOX) | 0.1303 | 0.1834 | -0.0177 |
| BALOFLOXACIN | 0.0928 | 0.1360 | -0.0169 |
| TOLFENAMIC ACID | 0.1003 | 0.1448 | -0.0166 |
| IVABRADINE HCL (PROCORALAN) | 0.1240 | 0.1738 | -0.0163 |
| AMIKACIN SULPHATE | 0.1143 | 0.1612 | -0.0158 |
| LAFUTIDINE | 0.1310 | 0.1819 | -0.0157 |
| LEUCOVORIN CALCIUM | 0.1317 | 0.1825 | -0.0156 |
| CREATININE | 0.1330 | 0.1839 | -0.0154 |
| TIOTROPIUM BROMIDE HYDRATE | 0.1314 | 0.1817 | -0.0152 |
| ARBIDOL HCL | 0.1188 | 0.1650 | -0.0144 |
| PHENYLEPHRINE HCL | 0.1310 | 0.1801 | -0.0143 |
| BROMHEXINE HCL | 0.1316 | 0.1802 | -0.0139 |
| NAFTIFINE HCL | 0.1260 | 0.1733 | -0.0138 |
| CONIVAPTAN HCL (VAPRISOL) | 0.1179 | 0.1632 | -0.0138 |
| PHENINDIONE (RECTADIONE) | 0.1275 | 0.1748 | -0.0136 |
| FLUBENDAZOLE (FLUTELMIUM) | 0.1510 | 0.2036 | -0.0133 |
| ENTECAVIR HYDRATE | 0.1340 | 0.1825 | -0.0133 |
| AMOXICILLIN SODIUM (AMOX) | 0.1282 | 0.1751 | -0.0132 |
| CORTISONE ACETATE (CORTONE) | 0.1356 | 0.1840 | -0.0129 |
| ACEMETACIN (EMFLEX) | 0.1254 | 0.1713 | -0.0128 |
| LOMEFLOXACIN HYDROCHLORIDE (MAXAQUIN) | 0.0378 | 0.0624 | -0.0126 |
| ENROFLOXACIN | 0.0412 | 0.0665 | -0.0125 |
| OXIBENDAZOLE | 0.1662 | 0.2212 | -0.0123 |
| DARIFENACIN HBR | 0.1226 | 0.1663 | -0.0116 |
| ESTRIOL | 0.1528 | 0.2037 | -0.0116 |
| CHLORPHENIRAMINE MALEATE | 0.1306 | 0.1760 | -0.0114 |
| METHYLTHIOURACIL | 0.1324 | 0.1774 | -0.0108 |
| MELOXICAM (MOBIC) | 0.1312 | 0.1751 | -0.0101 |
| ADEFOVIR DIPIVOXIL (PREVEON, HEPSERA) | 0.1310 | 0.1748 | -0.0101 |
| TETRACYCLINE HCL | 0.0751 | 0.1052 | -0.0098 |
| PROTIONAMIDE (PROTHIONAMIDE) | 0.1308 | 0.1742 | -0.0098 |
| NALTREXONE HCL | 0.1386 | 0.1835 | -0.0095 |
| CLEVIPREX (CLEVIDIPINE) | 0.1310 | 0.1737 | -0.0092 |
| TROSPIUM CHLORIDE (SANCTURA) | 0.1286 | 0.1707 | -0.0092 |
| GLIQUIDONE | 0.0980 | 0.1327 | -0.0091 |
| ESOMEPRAZOLE MAGNESIUM (NEXIUM) | 0.1329 | 0.1758 | -0.0090 |
| BROMPHENIRAMINE | 0.1300 | 0.1717 | -0.0086 |
| LONIDAMINE | 0.1094 | 0.1460 | -0.0085 |
| SULFADOXINE (SULPHADOXINE) | 0.1325 | 0.1747 | -0.0084 |
| PHENACETIN | 0.1341 | 0.1766 | -0.0084 |
| PHENOXYBENZAMINE HCL | 0.1436 | 0.1879 | -0.0080 |
| ARIPIPRAZOLE (ABILIFY) | 0.1252 | 0.1650 | -0.0079 |
| ALTRENOGEST | 0.1335 | 0.1748 | -0.0076 |
| PROPARACAINE HCL | 0.1279 | 0.1678 | -0.0075 |
| CLOXACILLIN SODIUM (CLOXACAP) | 0.1292 | 0.1689 | -0.0072 |
| MOEXIPRIL HCL | 0.1369 | 0.1784 | -0.0071 |
| STREPTOMYCIN SULFATE | 0.1211 | 0.1588 | -0.0071 |
| SUPROFEN (PROFENAL) | 0.1257 | 0.1645 | -0.0071 |
| MILNACIPRAN HCL | 0.1625 | 0.2100 | -0.0070 |
| METOPROLOL TARTRATE | 0.1274 | 0.1664 | -0.0069 |
| ETIDRONATE (DIDRONEL) | 0.1253 | 0.1635 | -0.0067 |
| KETOTIFEN FUMARATE (ZADITOR) | 0.1337 | 0.1738 | -0.0065 |
| EPALRESTAT | 0.1244 | 0.1621 | -0.0064 |
| SCOPINE | 0.1347 | 0.1747 | -0.0063 |
| DIMETHYL FUMARATE | 0.1316 | 0.1706 | -0.0061 |
| ADIPHENINE HCL | 0.1351 | 0.1746 | -0.0058 |
| AZATHIOPRINE (AZASAN, IMURAN) | 0.1338 | 0.1728 | -0.0056 |
| SITAFLOXACIN HYDRATE | 0.0088 | 0.0178 | -0.0056 |
| XYLOMETAZOLINE HCL | 0.1303 | 0.1681 | -0.0054 |
| TRIMEBUTINE | 0.1293 | 0.1669 | -0.0054 |
| DOMPERIDONE (MOTILIUM) | 0.1293 | 0.1667 | -0.0053 |
| EVISTA (RALOXIFENE HYDROCHLORIDE) | 0.1242 | 0.1604 | -0.0053 |
| CLOBETASOL PROPIONATE | 0.1305 | 0.1681 | -0.0051 |
| TIOPRONIN (THIOLA) | 0.1620 | 0.2069 | -0.0049 |
| AMITRIPTYLINE HCL | 0.1305 | 0.1676 | -0.0048 |
| (+,-)-OCTOPAMINE HCL | 0.1392 | 0.1782 | -0.0047 |
| MIZOLASTINE (MIZOLLEN) | 0.1362 | 0.1745 | -0.0047 |
| CILAZAPRIL MONOHYDRATE (INHIBACE) | 0.1367 | 0.1751 | -0.0046 |
| MITIGLINIDE CALCIUM | 0.1259 | 0.1615 | -0.0045 |
| MENADIONE | 0.1202 | 0.1545 | -0.0044 |
| ANTIPYRINE | 0.1355 | 0.1733 | -0.0044 |
| AZASETRON HCL (Y-25130) | 0.1347 | 0.1722 | -0.0043 |
| MIRTAZAPINE (REMERON, AVANZA) | 0.1255 | 0.1608 | -0.0043 |
| MEDROXYPROGESTERONE ACETATE | 0.1340 | 0.1712 | -0.0042 |
| TEMOCAPRIL HCL | 0.1340 | 0.1711 | -0.0041 |
| AZITHROMYCIN (ZITHROMAX) | 0.1243 | 0.1591 | -0.0041 |
| FLUOCINOLONE ACETONIDE (FLUCORT-N) | 0.1453 | 0.1847 | -0.0038 |
| ANAGRELIDE HCL | 0.1352 | 0.1720 | -0.0036 |
| HYDRALAZINE HYDROCHLORIDE | 0.1317 | 0.1673 | -0.0033 |
| GABEXATE MESYLATE | 0.1384 | 0.1752 | -0.0030 |
| BETAXOLOL HCL (BETOPTIC) | 0.1349 | 0.1707 | -0.0029 |
| RIBAVIRIN (COPEGUS) | 0.1286 | 0.1629 | -0.0028 |
| IDEBENONE | 0.1386 | 0.1752 | -0.0028 |
| DYCLONINE HCL | 0.1391 | 0.1756 | -0.0026 |
| ROFLUMILAST (DAXAS) | 0.1325 | 0.1672 | -0.0025 |
| MEGESTROL ACETATE | 0.1274 | 0.1608 | -0.0024 |
| 10-DAB (10-DEACETYLBACCATIN) | 0.1360 | 0.1713 | -0.0023 |
| SULBACTAM | 0.1267 | 0.1597 | -0.0022 |
| PANTOTHENIC ACID (PANTOTHENATE) | 0.1337 | 0.1683 | -0.0021 |
| PIOGLITAZONE HCL (ACTOS) | 0.1285 | 0.1618 | -0.0021 |
| METHSCOPOLAMINE (PAMINE) | 0.1291 | 0.1623 | -0.0019 |
| TRIPELENNAMINE HCL | 0.1255 | 0.1576 | -0.0017 |
| BENZTROPINE MESYLATE | 0.1355 | 0.1700 | -0.0017 |
| LISINOPRIL (ZESTRIL) | 0.1415 | 0.1774 | -0.0017 |
| CAPTOPRIL (CAPOTEN) | 0.1328 | 0.1662 | -0.0013 |
| CEFPROZIL HYDRATE (CEFZIL) | 0.1388 | 0.1735 | -0.0013 |
| SULFAMETHIZOLE (PROKLAR) | 0.1288 | 0.1611 | -0.0012 |
| MOXONIDINE | 0.1310 | 0.1637 | -0.0011 |
| ENOXACIN (PENETREX) | 0.1278 | 0.1597 | -0.0011 |
| RITODRINE HYDROCHLORIDE (YUTOPAR) | 0.1300 | 0.1620 | -0.0008 |
| ELTROMBOPAG (SB-497115-GR) | 0.0082 | 0.0109 | -0.0007 |
| NEOMYCIN SULFATE | 0.1280 | 0.1595 | -0.0006 |
| (R)-BACLOFEN | 0.1322 | 0.1645 | -0.0006 |
| LAPATINIB DITOSYLATE (TYKERB) | 0.1324 | 0.1647 | -0.0006 |
| QUINAPRIL HCL (ACCUPRIL) | 0.1333 | 0.1656 | -0.0004 |
| ARGATROBAN | 0.1260 | 0.1565 | -0.0003 |
| RIVASTIGMINE TARTRATE (EXELON) | 0.1438 | 0.1785 | -0.0003 |
| PAEONIFLORIN | 0.1360 | 0.1688 | -0.0003 |
| RIFAMPIN (RIFADIN, RIMACTANE) | 0.0074 | 0.0094 | -0.0001 |
| D-CYCLOSERINE | 0.1266 | 0.1570 | -0.0001 |
| DANOFLOXACIN MESYLATE | 0.0066 | 0.0082 | -0.0001 |
| CYPROHEPTADINE HCL (PERIACTIN) | 0.1483 | 0.1838 | 0.0000 |
| FLUNIXIN MEGLUMIN | 0.1246 | 0.1542 | 0.0001 |
| REBAMIPIDE | 0.1258 | 0.1556 | 0.0002 |
| TETRACAINE HYDROCHLORIDE (PONTOCAINE) | 0.1529 | 0.1891 | 0.0004 |
| WARFARIN | 0.1322 | 0.1634 | 0.0004 |
| PERINDOPRIL ERBUMINE (ACEON) | 0.1365 | 0.1685 | 0.0005 |
| DETOMIDINE HCL | 0.1369 | 0.1689 | 0.0006 |
| AMLODIPINE BESYLATE (NORVASC) | 0.1372 | 0.1690 | 0.0008 |
| CEPHALOMANNINE | 0.1443 | 0.1773 | 0.0012 |
| DISODIUM CROMOGLYCATE | 0.1459 | 0.1792 | 0.0012 |
| OXYBUTYNIN (DITROPAN) | 0.1370 | 0.1682 | 0.0012 |
| ALISKIREN HEMIFUMARATE | 0.1365 | 0.1675 | 0.0013 |
| FEXOFENADINE HCL | 0.1225 | 0.1502 | 0.0013 |
| BACITRACIN ZINC | 0.1363 | 0.1672 | 0.0014 |
| AMILORIDE HYDROCHLORIDE (MIDAMOR) | 0.1347 | 0.1651 | 0.0014 |
| FELBAMATE | 0.1435 | 0.1754 | 0.0019 |
| VITAMIN C (ASCORBIC ACID) | 0.1828 | 0.2236 | 0.0023 |
| EPINEPHRINE BITARTRATE (ADRENALINIUM) | 0.1371 | 0.1670 | 0.0023 |
| ABACAVIR SULFATE | 0.1377 | 0.1678 | 0.0023 |
| DL-ADRENALINE | 0.1365 | 0.1655 | 0.0029 |
| PREGNENOLONE | 0.1435 | 0.1742 | 0.0029 |
| AZACYCLONOL | 0.1349 | 0.1635 | 0.0030 |
| TYLOSIN TARTRATE | 0.0052 | 0.0025 | 0.0032 |
| RIFAXIMIN (XIFAXAN) | 0.0063 | 0.0038 | 0.0033 |
| MEPIVACAINE HCL | 0.1269 | 0.1531 | 0.0034 |
| PHENYTOIN SODIUM (DILANTIN) | 0.1361 | 0.1644 | 0.0034 |
| ROXITHROMYCIN (ROXL-150) | 0.0053 | 0.0023 | 0.0035 |
| STRONTIUM RANELATE (PROTELOS) | 0.1326 | 0.1597 | 0.0036 |
| L-ARGININE HCL | 0.1434 | 0.1732 | 0.0036 |
| FENOPROFEN CALCIUM HYDRATE | 0.1390 | 0.1676 | 0.0038 |
| DEFERASIROX (EXJADE) | 0.0626 | 0.0724 | 0.0042 |
| XYLAZINE HCL | 0.1383 | 0.1662 | 0.0042 |
| BUTOCONAZOLE NITRATE | 0.0054 | 0.0012 | 0.0044 |
| LOTEPREDNOL ETABONATE | 0.1429 | 0.1711 | 0.0048 |
| OLANZAPINE (ZYPREXA) | 0.1359 | 0.1617 | 0.0054 |
| UNKNOWN | 0.1368 | 0.1628 | 0.0054 |
| CICLOPIROX (PENLAC) | 0.1326 | 0.1575 | 0.0055 |
| PHENYTOIN (LEPITOIN) | 0.1325 | 0.1573 | 0.0055 |
| NILOTINIB (AMN-107) | 0.1588 | 0.1897 | 0.0057 |
| ALIBENDOL | 0.1357 | 0.1608 | 0.0059 |
| OXYTETRACYCLINE (TERRAMYCIN) | 0.1146 | 0.1345 | 0.0060 |
| ENTACAPONE | 0.1310 | 0.1546 | 0.0062 |
| PLERIXAFOR 8HCL (DB06809) | 0.1518 | 0.1803 | 0.0062 |
| DAUNORUBICIN HCL (DAUNOMYCIN HCL) | 0.0083 | 0.0023 | 0.0064 |
| TOLVAPTAN (OPC-41061) | 0.1488 | 0.1761 | 0.0067 |
| AVOBENZONE (PARSOL 1789) | 0.1331 | 0.1563 | 0.0070 |
| BESIFLOXACIN HCL (BESIVANCE) | 0.0089 | 0.0022 | 0.0071 |
| RIBOFLAVIN (VITAMIN B2) | 0.1375 | 0.1613 | 0.0073 |
| OTILONIUM BROMIDE | 0.0098 | 0.0028 | 0.0075 |
| SCOPOLAMINE HYDROBROMIDE | 0.1386 | 0.1621 | 0.0077 |
| ROXATIDINE ACETATE HCL | 0.1321 | 0.1539 | 0.0079 |
| CARBAZOCHROME SODIUM SULFONATE | 0.1476 | 0.1729 | 0.0081 |
| ECONAZOLE NITRATE (SPECTAZOLE) | 0.0090 | 0.0011 | 0.0081 |
| SOLIFENACIN SUCCINATE | 0.1405 | 0.1638 | 0.0082 |
| NAPHAZOLINE HYDROCHLORIDE (NAPHCON) | 0.1406 | 0.1640 | 0.0082 |
| CATHARANTHINE | 0.1453 | 0.1699 | 0.0083 |
| TRIMETAZIDINE DIHCL | 0.1417 | 0.1652 | 0.0084 |
| SULFAMETER (BAYRENA) | 0.1467 | 0.1714 | 0.0085 |
| LOVASTATIN (MEVACOR) | 0.1328 | 0.1540 | 0.0085 |
| MEPTAZINOL HCL | 0.1438 | 0.1676 | 0.0086 |
| ASPIRIN (ACETYLSALICYLIC ACID) | 0.1467 | 0.1711 | 0.0086 |
| OXFENDAZOLE | 0.1421 | 0.1651 | 0.0089 |
| IRSOGLADINE | 0.1276 | 0.1471 | 0.0089 |
| 2-THIOURACIL | 0.1416 | 0.1643 | 0.0090 |
| TRANEXAMIC ACID (TRANSAMIN) | 0.1344 | 0.1553 | 0.0091 |
| PREDNISOLONE ACETATE (OMNIPRED) | 0.1497 | 0.1739 | 0.0094 |
| RISEDRONIC ACID (ACTONEL) | 0.1402 | 0.1620 | 0.0094 |
| TRIFLUSAL | 0.1529 | 0.1777 | 0.0095 |
| RIMONABANT (SR141716) | 0.1470 | 0.1703 | 0.0096 |
| VILDAGLIPTIN (LAF-237) | 0.1456 | 0.1684 | 0.0097 |
| AZATADINE DIMALEATE | 0.1490 | 0.1721 | 0.0101 |
| FLUOCINONIDE (VANOS) | 0.1514 | 0.1748 | 0.0103 |
| UNKNOWN | 0.1397 | 0.1602 | 0.0103 |
| UNKNOWN | 0.1346 | 0.1539 | 0.0104 |
| GINKGOLIDE A | 0.1560 | 0.1803 | 0.0104 |
| AMIKACIN SULFATE | 0.1428 | 0.1639 | 0.0106 |
| ALVERINE CITRATE | 0.1431 | 0.1642 | 0.0106 |
| FENOPROFEN CALCIUM | 0.1379 | 0.1575 | 0.0108 |
| OSELTAMIVIR PHOSPHATE (TAMIFLU) | 0.1503 | 0.1729 | 0.0108 |
| BEPOTASTINE BESILATE | 0.1424 | 0.1627 | 0.0110 |
| APATINIB (YN968D1) | 0.1293 | 0.1461 | 0.0114 |
| GANCICLOVIR | 0.1354 | 0.1536 | 0.0115 |
| UNKNOWN | 0.1389 | 0.1578 | 0.0116 |
| VINPOCETINE (CAVINTON) | 0.1472 | 0.1680 | 0.0116 |
| PAROXETINE HCL | 0.1450 | 0.1652 | 0.0117 |
| ENALAPRIL MALEATE (VASOTEC) | 0.1334 | 0.1506 | 0.0119 |
| EMPTY | 0.1447 | 0.1645 | 0.0120 |
| PIMOBENDAN (VETMEDIN) | 0.1459 | 0.1657 | 0.0122 |
| TALC | 0.1324 | 0.1490 | 0.0122 |
| TIAMULIN HYD. PHSOP. | 0.1059 | 0.1160 | 0.0123 |
| PMSF (PHENYLMETHYLSULFONYL FLUORIDE) | 0.1536 | 0.1748 | 0.0125 |
| SUPLATAST TOSYLATE | 0.1449 | 0.1640 | 0.0125 |
| NALOXONE HCL | 0.1436 | 0.1619 | 0.0129 |
| BINDARIT | 0.1477 | 0.1669 | 0.0130 |
| INULIN | 0.1512 | 0.1711 | 0.0131 |
| PALONOSETRON HCL | 0.1456 | 0.1638 | 0.0135 |
| ROSUVASTATIN CALCIUM (CRESTOR) | 0.1650 | 0.1875 | 0.0136 |
| GENIPOSIDIC ACID | 0.1515 | 0.1708 | 0.0136 |
| NICORANDIL (IKOREL) | 0.1468 | 0.1649 | 0.0137 |
| CABAZITAXEL (JEVTANA) | 0.1354 | 0.1508 | 0.0137 |
| DILTIAZEM HCL (TIAZAC) | 0.1510 | 0.1698 | 0.0140 |
| PRAMIRACETAM | 0.1553 | 0.1750 | 0.0141 |
| CLAFEN (CYCLOPHOSPHAMIDE) | 0.1404 | 0.1559 | 0.0146 |
| CIPROFLOXACIN (CIPRO) | 0.1298 | 0.1427 | 0.0147 |
| ALLOPURINOL SODIUM (ALOPRIM) | 0.1450 | 0.1613 | 0.0148 |
| SULBACTAM SODIUM (UNASYN) | 0.1493 | 0.1663 | 0.0151 |
| TENOXICAM (MOBIFLEX) | 0.1435 | 0.1590 | 0.0151 |
| TROPICAMIDE | 0.1573 | 0.1760 | 0.0152 |
| PITOFENONE HCL | 0.1424 | 0.1575 | 0.0153 |
| AZELNIDIPINE | 0.1445 | 0.1600 | 0.0154 |
| LACIDIPINE (LACIPIL, MOTENS) | 0.1463 | 0.1616 | 0.0159 |
| URAPIDIL HCL | 0.1614 | 0.1802 | 0.0160 |
| CARBENICILLIN DISODIUM | 0.1362 | 0.1490 | 0.0160 |
| BUPIVACAINE HYDROCHLORIDE (MARCAIN) | 0.1409 | 0.1548 | 0.0160 |
| ZONISAMIDE | 0.1425 | 0.1565 | 0.0162 |
| PROBUCOL | 0.1532 | 0.1696 | 0.0163 |
| TIOXOLONE | 0.1534 | 0.1698 | 0.0164 |
| ORNIDAZOLE | 0.1428 | 0.1565 | 0.0164 |
| ELLAGIC ACID | 0.1550 | 0.1716 | 0.0165 |
| ATAZANAVIR SULFATE | 0.1552 | 0.1713 | 0.0169 |
| PAZOPANIB HCL | 0.1505 | 0.1648 | 0.0175 |
| ULIPRISTAL | 0.1475 | 0.1610 | 0.0175 |
| IBANDRONATE SODIUM | 0.1506 | 0.1646 | 0.0178 |
| LURASIDONE HCL | 0.1398 | 0.1512 | 0.0178 |
| ROCURONIUM BROMIDE | 0.1573 | 0.1726 | 0.0180 |
| 5-AMINOLEVULINIC ACID HYDROCHLORIDE | 0.1408 | 0.1518 | 0.0182 |
| BETA CAROTENE | 0.1540 | 0.1681 | 0.0183 |
| UBENIMEX (BESTATIN) | 0.1531 | 0.1668 | 0.0185 |
| CISATRACURIUM BESYLATE (NIMBEX) | 0.1549 | 0.1690 | 0.0185 |
| MOMETASONE FUROATE | 0.1492 | 0.1619 | 0.0185 |
| LORNOXICAM (XEFO) | 0.1345 | 0.1437 | 0.0185 |
| CLINDAMYCIN PHOSPHATE | 0.1448 | 0.1563 | 0.0186 |
| NYSTATIN (MYCOSTATIN) | 0.1532 | 0.1667 | 0.0186 |
| CYTARABINE | 0.1464 | 0.1582 | 0.0187 |
| PERAMIVIR TRIHYDRATE | 0.1559 | 0.1697 | 0.0189 |
| GIMERACIL | 0.1661 | 0.1822 | 0.0190 |
| MOCLOBEMIDE | 0.1502 | 0.1625 | 0.0190 |
| AZILSARTAN MEDOXOMIL (TAK-491) | 0.1391 | 0.1487 | 0.0191 |
| RASAGILINE MESYLATE | 0.1602 | 0.1746 | 0.0192 |
| FORMOTEROL HEMIFUMARATE | 0.1398 | 0.1487 | 0.0197 |
| NORADRENALINE BITARTRATE MONOHYDRATE (LEVOPHED) | 0.1547 | 0.1672 | 0.0197 |
| TRAZODONE HYDROCHLORIDE (DESYREL) | 0.1551 | 0.1674 | 0.0200 |
| BLONANSERIN (LONASEN) | 0.1567 | 0.1692 | 0.0201 |
| RISEDRONATE SODIUM | 0.1556 | 0.1677 | 0.0203 |
| BUFLOMEDIL HCL | 0.1575 | 0.1700 | 0.0203 |
| ERDOSTEINE | 0.1617 | 0.1751 | 0.0205 |
| BENDAMUSTINE HCL | 0.1556 | 0.1675 | 0.0205 |
| LINAGLIPTIN (BI-1356) | 0.1536 | 0.1646 | 0.0207 |
| MEDETOMIDINE HCL | 0.1511 | 0.1613 | 0.0209 |
| RACTOPAMINE HCL | 0.1520 | 0.1621 | 0.0211 |
| IDOXURIDINE | 0.1435 | 0.1515 | 0.0213 |
| FESOTERODINE FUMARATE (TOVIAZ) | 0.1747 | 0.1900 | 0.0213 |
| BUFEXAMAC | 0.1536 | 0.1638 | 0.0214 |
| ROSIGLITAZONE MALEATE | 0.1401 | 0.1466 | 0.0218 |
| MEMANTINE HCL (NAMENDA) | 0.1696 | 0.1828 | 0.0220 |
| PEFLOXACIN MESYLATE | 0.0359 | 0.0171 | 0.0221 |
| DROXIDOPA (L-DOPS) | 0.1517 | 0.1605 | 0.0222 |
| TOLBUTAMIDE | 0.1419 | 0.1481 | 0.0224 |
| ONDANSETRON HCL (ZOFRAN) | 0.1472 | 0.1546 | 0.0224 |
| DICHLORPHENAMIDE (DICLOFENAMIDE) | 0.1716 | 0.1844 | 0.0228 |
| ATROPINE | 0.1570 | 0.1662 | 0.0228 |
| ASPARTAME | 0.1546 | 0.1631 | 0.0229 |
| MOGUISTEINE | 0.1673 | 0.1788 | 0.0230 |
| MOSAPRIDE CITRATE | 0.1581 | 0.1674 | 0.0231 |
| DAPTOMYCIN | 0.1484 | 0.1552 | 0.0231 |
| CEFDITOREN PIVOXIL | 0.1496 | 0.1566 | 0.0232 |
| PRANOPROFEN | 0.1463 | 0.1526 | 0.0232 |
| ADENINE | 0.1491 | 0.1549 | 0.0241 |
| DOCETAXEL (TAXOTERE) | 0.1607 | 0.1690 | 0.0243 |
| LAMOTRIGINE | 0.1624 | 0.1709 | 0.0244 |
| PHENTOLAMINE MESILATE | 0.1546 | 0.1612 | 0.0245 |
| CHLORMEZANONE (TRANCOPAL) | 0.1482 | 0.1528 | 0.0249 |
| GENIPOSIDE | 0.1675 | 0.1764 | 0.0251 |
| NILVADIPINE (ARC029) | 0.1607 | 0.1677 | 0.0253 |
| GEMCITABINE HCL (GEMZAR) | 0.0319 | 0.0077 | 0.0256 |
| THIOGUANINE | 0.1498 | 0.1536 | 0.0258 |
| CYTIDINE | 0.1549 | 0.1598 | 0.0259 |
| VALACICLOVIR HCL | 0.1565 | 0.1617 | 0.0260 |
| CLOMIFENE CITRATE (SEROPHENE) | 0.0401 | 0.0170 | 0.0264 |
| ARTICAINE HCL | 0.1369 | 0.1363 | 0.0269 |
| ALTRETAMINE (HEXALEN) | 0.1694 | 0.1765 | 0.0270 |
| MEGLUMINE | 0.1585 | 0.1627 | 0.0273 |
| NISOLDIPINE (SULAR) | 0.1671 | 0.1732 | 0.0273 |
| CANDESARTAN CILEXETIL (ATACAND) | 0.0415 | 0.0171 | 0.0277 |
| ROFECOXIB (VIOXX) | 0.1593 | 0.1622 | 0.0284 |
| ESTRADIOL VALERATE | 0.1560 | 0.1579 | 0.0285 |
| LEVOSIMENDAN | 0.1545 | 0.1554 | 0.0291 |
| AZILSARTAN (TAK-536) | 0.1546 | 0.1545 | 0.0299 |
| THALIDOMIDE | 0.1607 | 0.1610 | 0.0307 |
| IRINOTECAN | 0.1660 | 0.1672 | 0.0310 |
| BIFONAZOLE | 0.0385 | 0.0090 | 0.0312 |
| MICONAZOLE NITRATE | 0.0389 | 0.0088 | 0.0318 |
| SODIUM SALICYLATE | 0.1656 | 0.1656 | 0.0320 |
| CINEPAZIDE MALEATE | 0.1614 | 0.1596 | 0.0325 |
| BETAHISTINE 2HCL | 0.1699 | 0.1701 | 0.0326 |
| BETAMETHASONE DIPROPIONATE (DIPROLENE) | 0.1686 | 0.1683 | 0.0327 |
| OSI-420 (DESMETHYL ERLOTINIB) | 0.1573 | 0.1537 | 0.0332 |
| AZLOCILLIN SODIUM SALT | 0.1576 | 0.1540 | 0.0333 |
| METRONIDAZOLE (FLAGYL) | 0.1649 | 0.1627 | 0.0335 |
| METHYLPREDNISOLONE | 0.1624 | 0.1589 | 0.0342 |
| RIMANTADINE (FLUMADINE) | 0.1568 | 0.1513 | 0.0347 |
| AMIDOPYRINE | 0.1674 | 0.1644 | 0.0347 |
| FLUTICASONE PROPIONATE (FLONASE, VERAMYST) | 0.1578 | 0.1525 | 0.0347 |
| CLARITHROMYCIN (BIAXIN, KLACID) | 0.0437 | 0.0094 | 0.0361 |
| SULPHADIMETHOXINE | 0.1545 | 0.1459 | 0.0368 |
| TAURINE | 0.1643 | 0.1562 | 0.0382 |
| ORPHENADRINE CITRATE (NORFLEX) | 0.1769 | 0.1706 | 0.0392 |
| CLINDAMYCIN PALMITATE HCL | 0.1150 | 0.0933 | 0.0397 |
| GENIPIN | 0.1803 | 0.1737 | 0.0401 |
| AMIKACIN HYDRATE | 0.1717 | 0.1622 | 0.0408 |
| NIMESULIDE | 0.1445 | 0.1268 | 0.0421 |
| FLUMEQUINE | 0.0753 | 0.0403 | 0.0428 |
| OZAGREL HCL | 0.1696 | 0.1551 | 0.0443 |
| ATOVAQUONE (ATAVAQUONE) | 0.1790 | 0.1666 | 0.0445 |
| ETHISTERONE | 0.1790 | 0.1663 | 0.0448 |
| SULFATHIAZOLE | 0.1803 | 0.1617 | 0.0498 |
| AMPICILLIN SODIUM | 0.1356 | 0.1008 | 0.0542 |
| AMOXICILLIN (AMOXYCILLIN) | 0.1485 | 0.1144 | 0.0562 |
| PROCARBAZINE HCL (MATULANE) | 0.1589 | 0.1241 | 0.0587 |
| BEXAROTENE | 0.0639 | 0.0023 | 0.0620 |
| CLORSULON | 0.2017 | 0.1664 | 0.0674 |
| THIABENDAZOLE | 0.2099 | 0.1751 | 0.0686 |
| PROPYLTHIOURACIL | 0.1627 | 0.1144 | 0.0704 |
| CURCUMIN | 0.1497 | 0.0910 | 0.0763 |
| VANCOMYCIN HCL (VANCOCIN) | 0.0889 | 0.0039 | 0.0857 |
| ABIRATERONE ACETATE (CB7630) | 0.1393 | 0.0140 | 0.1280 |
| OXACILLIN SODIUM MONOHYDRATE | 0.1342 | 0.0021 | 0.1325 |
| TEBIPENEM PIVOXIL (L-084) | 0.1760 | 0.0022 | 0.1742 |
