# Supplementary figures and images for: Repurposing drugs with specific activity against L-form bacteria
Source: Front Microbiol. 2023 Apr 4;14:1097413. doi: 10.3389/fmicb.2023.1097413 (PMC10110866; doi:10.3389/fmicb.2023.1097413)

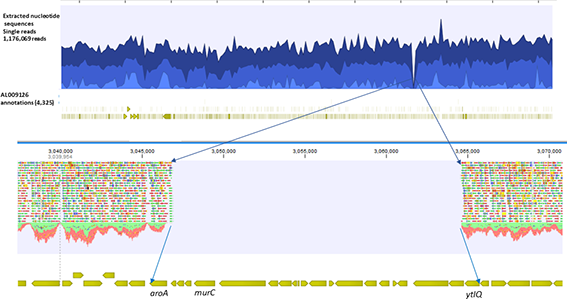

Supplement: Supplementary Figure 1 — Schematic representation of the chromosomal region deleted in strain 18. The relative position of the deleted 18 Kbp compared to B. subtilis reference genome (AL009126, https://www.ncbi.nlm.nih.gov/genbank/) is shown. The data was generated using Qiagen CLC Bio software. [file Image_1.TIF]

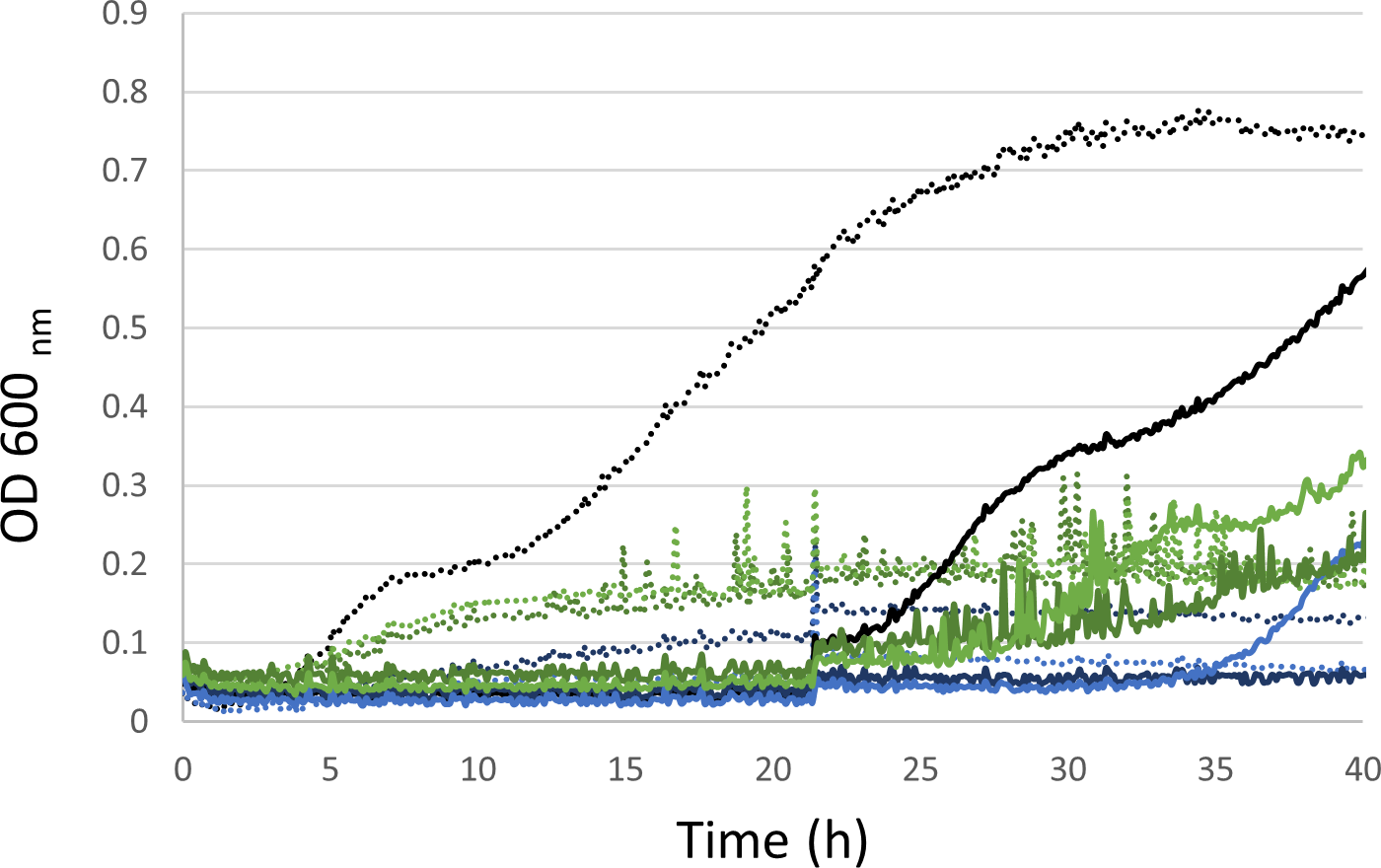

Supplement: Supplementary Figure 2 — Test for recovery of L-form growth after treatment with specific inhibitors. L-form strain Δ18 was grown in MSM/NB medium for 22 h at 30°C, then the culture was diluted and aliquots of the culture were treated with 5 or 20 μM of manidipine (light green and dark green, respectively) or flunarizine (light and dark blue), or a similar volume (0.5 μL) of DMSO only (black), to give a total volume of 200 μL in the wells of a 96 well plate (rows A to D). Growth (OD600) in each well (dotted lines) was recorded continuously. After 22 h a 10 μL sample of each culture was diluted into 190 μL pre warmed fresh medium with no compound addition (rows E to H). Culture growth was then continued for a further 20 h. Colors are as above but with solid lines. Note that the solid line values prior to dilution show the background levels of absorbance for empty wells. Each row contained three replicates and plots shown are averages of the three. [file Image_2.TIF]
